# Physics-Informed Artificial Intelligence Design of Picomolar Nanobodies Enables Deep Tumor Penetration and High-Contrast Imaging

**DOI:** 10.34133/research.1325

**Published:** 2026-06-24

**Authors:** Ning Shi, Caiping Ren, Liang Zhang, Lei Wang, Xuechen Yang, Xiaobo Li, Yangyihua Zhou, Jie Wang, Pinnan Zhao, Chaoyan Yao, Yaowei Ma, Juan Tian, Qianping Huang, Can Xu, Xiaonan Kuang, Weidong Liu, Xingjun Jiang, Jun Ye, Xiang Gao, Longlong Luo

**Affiliations:** ^1^ Department of Neurosurgery, Xiangya Hospital, Xiangya School of Basic Medical Science, Central South University, Changsha, Hunan 410008, China.; ^2^ Academy of Military Medical Sciences, Beijing 100850, China.; ^3^ NHC Key Laboratory of Carcinogenesis, The Key Laboratory of Carcinogenesis and Cancer Invasion of the Chinese Ministry of Education, Cancer Research Institute, Xiangya School of Basic Medical Science, Central South University, Changsha, Hunan 410078, China.; ^4^ National Clinical Research Center for Geriatric Disease, Xiangya Hospital, Central South University, Changsha, Hunan 410008, China.; ^5^ Hunan Normal University School of Medicine, Changsha, Hunan 410081, China.; ^6^ Beijing Key Laboratory of Key Technologies for Natural Drug Delivery and Novel Formulations, Institute of Materia Medica, Chinese Academy of Medical Sciences & Peking Union Medical College, Beijing 100050, China.

## Abstract

The clinical utility of nanobodies in solid tumor therapy is constrained by a fundamental biophysical trade-off: rapid renal clearance necessitates half-life extension, which in turn demands ultrahigh affinity to prevent dissociation from the target under systemic washout conditions. While generative artificial intelligence has substantially advanced structure prediction, it often fails to resolve the subtle energetic frustrations at protein–protein interfaces required for affinity maturation. Here, we present a physics-informed artificial intelligence framework that integrates AlphaFold 3 structural priors with molecular dynamics simulations to rationally design a picomolar anti-carcinoembryonic antigen nanobody. By employing variable dielectric molecular mechanics/generalized Born surface area decomposition, we identified interfacial residues that were structurally permissible but thermodynamically suboptimal. We subsequently constructed a focused library to resolve these bottlenecks through electrostatic optimization, desolvation penalty minimization, and van der Waals packing refinement. This strategy achieved a 99% binding positivity rate and yielded variants with picomolar affinity (*K_D_* ≈ 44 pM)—an ~306-fold improvement over the parental clone—without compromising thermal stability (*T*_m_ > 63 °C). To translate these biophysical gains into therapeutic efficacy, we engineered bispecific nanobodies fusing the affinity-matured domains with an anti-human serum albumin binder. In vivo longitudinal imaging of colorectal cancer xenografts revealed a “lock-and-hold” phenotype, characterized by deep intratumoral penetration and sustained retention (>168 h). This work demonstrates that coupling geometric deep learning with rigorous physical principles overcomes the inefficiencies of stochastic screening, providing a valuable framework that may be adapted for the rational development of high-potency biologics across various therapeutic targets.

## Introduction

Carcinoembryonic antigen (CEA, also designated as CEACAM5), a classic cell surface glycoprotein belonging to the carcinoembryonic antigen-related cell adhesion molecule (CEACAM) family, stands out as one of the most extensively used tumor markers for precision oncology and molecular imaging [1,2]. While conventional high-affinity monoclonal antibodies remain a cornerstone of targeted cancer therapy [3], their bulky architecture (~150 kDa) frequently restricts them to the perivascular space in solid tumors—a phenomenon classically known as the “binding site barrier” (BSB) [4–6]. In contrast, nanobodies (VHHs), single-domain fragments derived from Camelidae heavy-chain antibodies, offer unique structural advantages to overcome this physical limitation [7–10]. Their diminutive footprint (~15 kDa) and extended CDR3 loops allow them to rapidly percolate through dense stromal barriers and bind cryptic epitopes often inaccessible to conventional immunoglobulins [11,12].

However, the clinical translation of VHHs as high-contrast imaging probes is severely challenged by rapid renal clearance and suboptimal plasma half-life [10]. To mitigate this, half-life extension strategies, such as genetic fusion with an anti-human serum albumin (anti-HSA) binder, are frequently employed [13,14]. Yet, this strategy unmasks a critical dependency on binding thermodynamics: merely extending systemic circulation is insufficient if the tumor-targeting moiety dissociates rapidly. To successfully convert prolonged blood exposure into sustained, high-contrast tumor retention against interstitial washout, the probe must possess ultrahigh binding affinity. Consequently, rational affinity maturation from the nanomolar to the picomolar regime is a fundamental prerequisite to optimize the pharmacokinetic (PK)/pharmacodynamic profiles and unlock their full diagnostic potential [15].

Despite this necessity, efficient affinity maturation remains a major bottleneck. Traditional experimental approaches, such as directed evolution via error-prone polymerase chain reaction (PCR) or DNA shuffling, rely on stochastic mutagenesis to navigate sequence space. These methods are inherently labor-intensive, necessitating the screening of high-complexity libraries (often exceeding 10^9^ variants) and frequently introducing destabilizing mutations [16,17]. Conversely, traditional computational antibody design (CADD) has been historically limited by structural and energetic inaccuracies. Homology modeling often fails to capture the conformational plasticity of hypervariable loops, while static scoring functions struggle to correlate with experimental binding free energies due to inadequate treatment of solvation effects and backbone flexibility [18,19].

The integration of deep learning with physics-based simulations offers a robust solution to these challenges. Recent computational advancements have driven major breakthroughs in this field; for instance, while emerging sequence–structure pre-trained language models (e.g., the S^2^ALM foundation model, which integrates 1-dimensional sequence and 3-dimensional structural data to accurately predict antigen–antibody binding affinities) [20] have demonstrated remarkable potential in capturing comprehensive antibody representations, tools like AlphaFold 3 provide highly accurate structural priors even without crystallographic templates [21,22]. However, while these tools provide reliable structural priors, they cannot fully resolve the dynamic quaternary arrangement of antibody–antigen complexes or accurately quantify the energetic contributions of interfacial residues. The critical distinction of our physics-informed artificial intelligence (AI) framework lies in its ability to transcend the “structure–energy gap” that limits conventional AI design. While structural predictors like AlphaFold 3 provide high-fidelity backbone snapshots, they are inherently limited in quantifying the dynamic free energy of binding (Δ*G*_bind_) [21–23]. Traditional CADD models often struggle with “false-positive” residues that exhibit favorable spatial complementarity but incur a high energetic cost upon desolvation. By integrating molecular dynamics (MD)-based ensemble sampling with vector-decomposition (variable dielectric molecular mechanics/generalized Born surface area [vd-MM/GBSA]) analysis, our pipeline provides the thermodynamic resolution necessary to identify residues that are structurally permissible but energetically frustrated. To address this, we employ a hybrid computational strategy: molecular docking is used to sample the binding interface, followed by MD simulations and MM/GBSA calculations [24–26]. This physics-based approach captures dynamic flexibility, enabling the rigorous identification of “thermodynamic hotspots”—residues that are structurally permissible but energetically suboptimal—thereby defining precise coordinates for rational optimization [24,27].

In this study, we aimed to perform rational affinity maturation on a moderate-affinity anti-CEA nanobody (parental Nb, *K_D_* ≈ 13.5 nM) and to systematically validate the direct impact of enhanced affinity on in vivo tumor-targeting efficacy. We present an integrated framework combining geometric deep learning with thermodynamic refinement to guide nanobody engineering. Our workflow proceeds through a systematic cascade: (a) generation of high-confidence structural models using AlphaFold 3 [21], (b) de novo reconstruction of the complex via ensemble docking, (c) identification of energetic bottlenecks through MD-based decomposition analysis [27], and (d) construction of a focused library targeting the interfacial microenvironment. By coupling this in silico design with phage display, we evolved a parental nanobody to picomolar affinity (*K_D_* ≈ 44 pM), achieving an ~306-fold improvement in binding strength. Furthermore, we engineered a bispecific antibody (bsAb) using these variants, which demonstrated superior tumor uptake and prolonged retention in vivo. While recent comparative studies on full-length bsAbs emphasize that complex spatial architectures often introduce steric hindrance and unpredictable pharmacokinetics [28], our modular nanobody fusion circumvents these format-related limitations, achieving true functional autonomy. This work establishes a potentially scalable and high-efficiency workflow that facilitates the rational design of potent biologics [21,29,30].

## Results

### Structure-guided epitope mapping and physics-informed rational design of a focused nanobody library

**Fig. 1.**
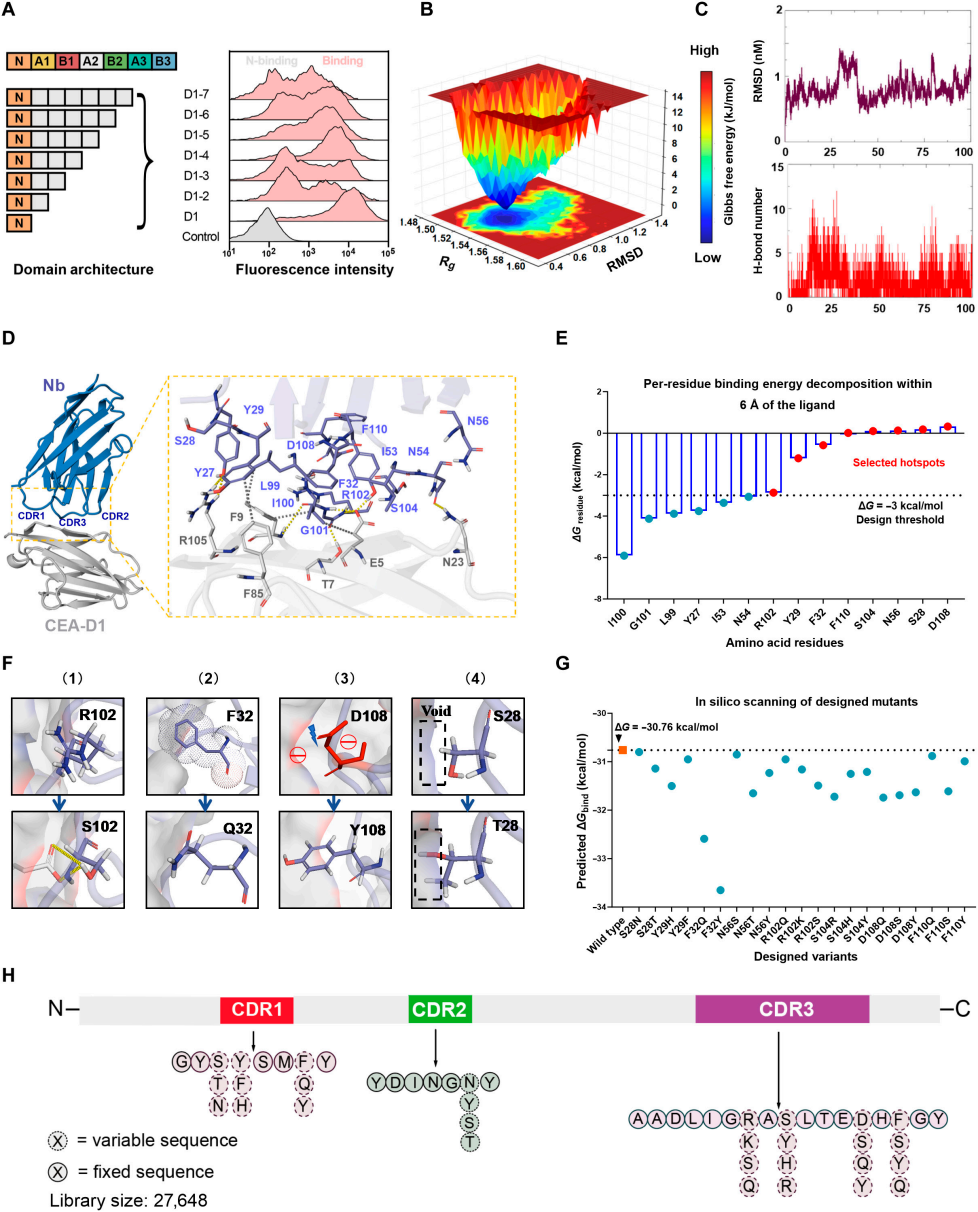
Structure-guided epitope mapping and physics-informed rational design enable targeted affinity maturation of the anti-carcinoembryonic antigen (anti-CEA) nanobody. (A) Domain-level epitope mapping via cell surface display. Schematic illustration (left) and representative flow cytometry histograms (right) localize the CE8 binding site specifically to the N-terminal D1 domain of CEA. (B) Gibbs free energy landscape (FEL) analysis projected onto the radius of gyration *R_g_* and root-mean-square deviation (RMSD). The deep energy basin indicates a stable conformational state for the Nb–CEA complex. (C) Molecular dynamics (MD) simulation trajectories (100 ns) monitoring backbone RMSD stability (top) and the dynamic maintenance of interfacial hydrogen bonds (bottom). (D) Structural snapshot of the Nb–CEA interface at atomic resolution, highlighting key residues and the network of noncovalent interactions mediating binding. (E) Per-residue binding free energy decomposition (Δ*G*_residue_). Red dots indicate residues identified as targets for maturation based on an energetic threshold (contribution > −3 kcal/mol). (F) Schematic of the 4 primary rational design strategies employed: (1) electrostatic/H-bond enhancement, (2) solvation optimization, (3) charge/packing optimization, and (4) repair of interfacial packing defects (cavity filling). (G) In silico prediction of relative binding affinity changes (Δ*G*) for designed variants. The wild-type (WT) reference is marked by an orange square; lower values indicate predicted affinity improvement. (H) Strategy for focused library construction. The “variable sequence” notation denotes positions randomized with specific amino acid mixtures derived from the computational design output.

To deconstruct the structural determinants of CE8 recognition, we first delineated its minimal binding interface. Although full-length CEA presents a multidomain architecture, cell surface display of truncation variants unambiguously localized the CE8 epitope to the N-terminal immunoglobulin variable (IgV)-like domain (D1) (Fig. 1A and Fig. S1A). This experimental anchoring was pivotal, as it substantially reduced the conformational search space, thereby facilitating high-resolution modeling.

Leveraging this constraint, we generated high-confidence structural models of the CEA-D1 antigen and the CE8 nanobody using AlphaFold 3. The predicted interface exhibited exceptional structural integrity, evidenced by predicted template modeling scores of 0.88 and 0.86, respectively, and consistently high predicted local distance difference test scores (>90) across the core β-sandwich framework (Fig. S1B). To rigorously validate the thermodynamic viability of this in silico model, we performed consensus docking using the HADDOCK2.4 docking platform. Among the 100 generated poses, the selected conformation (pose 1) achieved the highest composite score across AMBER, AMOEBA, and DFIRE force fields, indicating a globally optimal binding mode (Fig. S1C).

To further assess the dynamic stability and physical validity of the predicted interface, the complex was subjected to a 100-ns MD simulation. Gibbs free energy landscape analysis revealed that the complex resides in a deep, thermodynamically robust energy basin (Fig. 1B). The trajectories exhibited a stable root-mean-square deviation and sustained interfacial hydrogen bonds (averaging ~6), confirming a stable binding conformation devoid of appreciable drift (Fig. 1C). Additional MD metrics, including radius of gyration (*R_g_*), solvent-accessible surface area, and per-residue root-mean-square fluctuation, further corroborated the structural compactness and local flexibility required for paratope optimization (Fig. S1D to F).

To transcend the limitations of static structural snapshots and capture the thermodynamic signatures often missed by generative AI models, we performed a granular thermodynamic profiling of the complex using vd-MM/GBSA decomposition. Focusing on the immediate interaction shell, we identified 14 amino acid residues on the antibody located within 6 Å of the antigen interface (Fig. 1D). This analysis dissected the total binding free energy into individual residue contributions, exposing specific energetic frustrations—interfacial residues that contributed suboptimally (energies > −3 kcal/mol) to the binding enthalpy (Fig. 1E). Notably, these frustrated residues often appear structurally compatible in static predictors like AlphaFold 3, yet they represent thermodynamic bottlenecks due to suboptimal local environments. Guided by this quantitative threshold, we pinpointed 8 specific residues (S28, Y29, F32, N56, R102, S104, D108, and F110) as weak anchors requiring optimization.

To resolve these bottlenecks, we formulated a rational design strategy termed “interfacial microenvironment optimization” addressing 4 precise physical mechanisms typically overlooked by traditional CADD or static AI scoring (Fig. 1F): (1) Electrostatic and directional H-bond refinement: Exemplified by the R102S mutation, where replacing a bulky arginine with a polar serine eliminates stereochemical hindrance and establishes a directional hydrogen-bond network. Unlike static docking, our approach optimizes these interactions across conformational ensembles, satisfying specific orientation dependencies that govern binding stability. (2) Mitigation of desolvation penalties: As seen in the F32Q mutation, we substituted a hydrophobic phenylalanine with a polar glutamine to reduce the desolvation cost—the energetic penalty of removing water molecules from the interface during binding. (3) Charge neutralization and aromatic networking: Illustrated by the D108Y mutation, where replacing an acidic aspartate neutralizes localized electrostatic repulsion while introducing stabilizing π–π stacking interactions, effectively “locking” the interface thermodynamically. (4) Dynamic shape complementarity: The S28T mutation utilizes an additional methyl group to fill transient interfacial voids. These defects, identified through MD trajectories, represent “breathing” cavities that are invisible to static structural snapshots but critical for achieving optimal packing density.

Building on these biophysical principles, we performed in silico saturation mutagenesis at the 8 identified hotspots to calculate the binding free energy of potential variants relative to the wild-type complex (Δ*G*_bind_ approximately −30.76 kcal/mol). Detailed thermodynamic decomposition confirmed that these affinity gains stemmed from the targeted optimization of electrostatic and solvation terms (Table S1). In this screening process, any mutation exhibiting a binding free energy lower than that of the wild type was selected as a potential affinity-enhancing candidate (Fig. 1G). To bridge computational prediction with biological reality, these high-scoring variants were integrated into a combinatorial focused library. By permuting the selected mutations across the 8 targeted positions within CDR1, CDR2, and CDR3, we generated a library containing 27,648 unique sequence variants (Fig. 1H). This strategy created a computationally compressed search space that retained the diversity of predicted mutations, enabling the experimental interrogation of epistatic interactions that isolated predictions might otherwise overlook.

### Construction and rigorous quality control of the structure-guided focused library

**Fig. 2.**
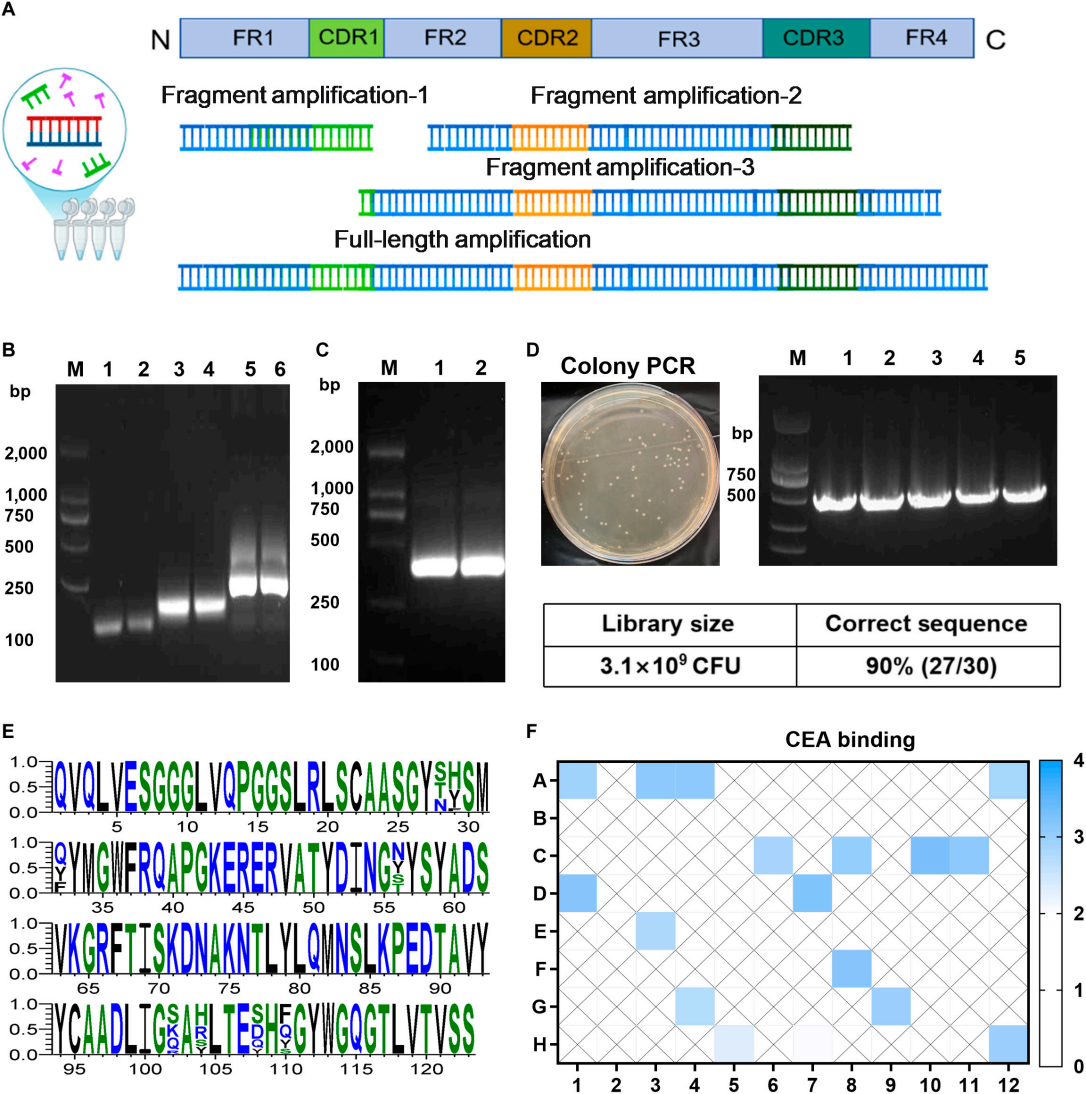
Construction and comprehensive quality control of the focused nanobody library. (A) Schematic illustration of the multistep overlap extension polymerase chain reaction (OE-PCR) strategy employed for precise library assembly. The process involves the parallel amplification of framework (FR) and complementarity-determining regions (CDRs) followed by full-length gene reconstruction. (B and C) Electrophoretic verification of stepwise library assembly. (B) Analysis of intermediate fragments: Lanes 1 and 2 show fragment 1 (FR1-CDR1), lanes 3 and 4 show fragment 2 (FR2-CDR2), and lanes 5 and 6 show fragment 3 (FR3-CDR3). (C) Confirmation of the final full-length VHH gene assembly (~400 bp, lanes 1 and 2). M: DNA molecular weight marker. (D) Quantitative assessment of library quality. The panel displays a representative colony PCR gel (right), the total library size (3.1 × 10^9^ CFU), and the sequence fidelity rate (90%, 27/30 correct clones) determined via Sanger sequencing. (E) Sequence logo analysis derived from randomly selected library clones. The plot confirms the correct incorporation of designed amino acid diversity at specific hotspot positions within the CDRs. (F) Heatmap of carcinoembryonic antigen (CEA)-binding activity for individual clones randomly selected from the constructed focused library. Binding strength was assessed via monoclonal enzyme-linked immunosorbent assay (ELISA) (OD_450_). The distribution of high-affinity binders (blue) versus nonbinders (crossed-out/white) demonstrates the high functional rate achieved through the rational design strategy.

To accurately translate the in silico design into a physical library, we employed a precision-guided, multistep overlap extension PCR strategy (Fig. 2A). During the construction of the focused library, we strictly followed AI-guided design principles and performed precise degenerate codon design for the 8 pre-identified hotspots to achieve efficient coverage of the target amino acids. A zero-redundancy strategy was adopted at each mutation site: via customized primer mixing, every clone generated was ensured to harbor the designed target mutations, while by-products were completely eliminated. The detailed degenerate codon scheme is summarized in Table S2. Unlike traditional error-prone PCR which introduces mutations randomly, this approach allowed for the targeted introduction of degenerate codons specifically at the 8 identified thermodynamic hotspots. The assembly process was rigorously monitored: agarose gel electrophoresis confirmed the clean amplification of individual gene fragments (Fig. 2B) and their successful assembly into a single, distinct band at approximately 400 bp, corresponding to the full-length VHH gene (Fig. 2C).

Following electroporation into *Escherichia coli* TG1 cells, the library size was determined to be 3.1 × 10^9^ CFU by serial dilution plating. To assess the quality of this massive repertoire, we randomly selected 30 clones for colony PCR and Sanger sequencing. The results revealed a high assembly efficiency, with 90% (27/30) of the clones containing the correct full-length insert and no frameshift mutations (Fig. 2D). Furthermore, sequence logo analysis of the randomized regions demonstrated that the amino acid distribution closely mirrored the theoretical design (Fig. 2E), confirming that the chemical diversity of the primer pool was effectively transferred to the biological library without appreciable bias.

Finally, to verify the functional integrity of the library prior to selection, we performed a preliminary monoclonal enzyme-linked immunosorbent assay (ELISA) screening on 96 randomly picked clones from the unselected library (Fig. 2F), which revealed an initial binding positivity rate of 16.7% (16/96). This initial hit rate is substantially higher than the typical values observed in stochastic mutagenesis libraries (often <0.1%), providing direct evidence that our physics-informed design effectively pre-enriched the library with functional variants. By filtering out energetically unfavorable sequences *in silico*, we successfully compressed the search space by several orders of magnitude, allowing for the rapid isolation of picomolar binders from a compact library of only 2.7 × 10^4^ variants.

### Phage display screening and identification of high-affinity variants

**Fig. 3.**
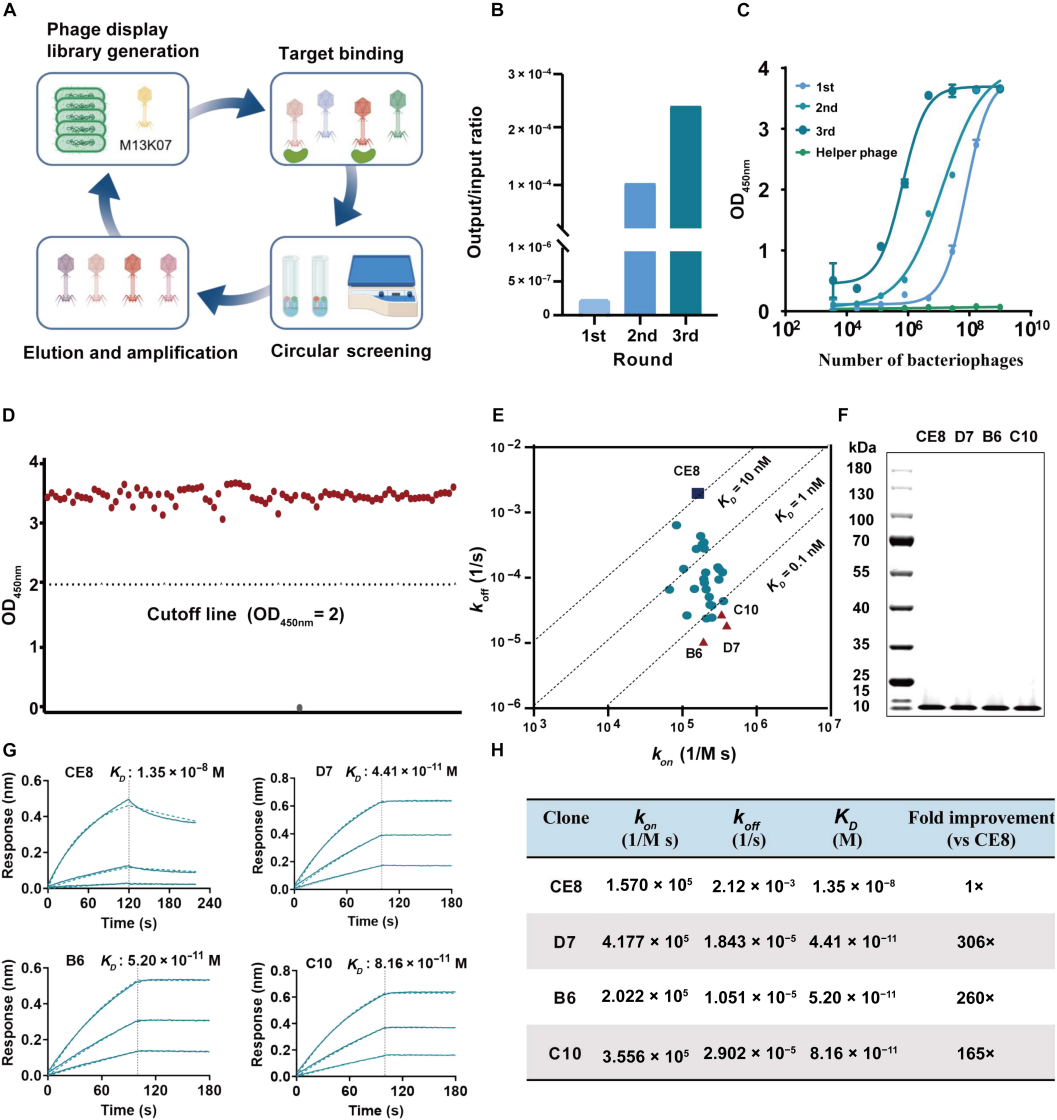
Phage display selection and characterization of high-affinity nanobody variants. (A) Schematic illustration of the competitive phage display panning strategy used to select high-affinity binders against carcinoembryonic antigen (CEA). (B) Enrichment efficiency over 3 rounds of panning. The bar chart displays the ratio of output to input phage titers, indicating pronounced enrichment of specific binders. (C) Polyclonal phage enzyme-linked immunosorbent assay (ELISA) showing the progressive increase in CEA-binding activity of the amplified phage pool from the first (1st) to the third (3rd) round. (D) Monoclonal phage ELISA of 96 randomly picked clones from the third-round output. The scatter plot represents the binding signal (OD_450nm_) of individual clones to CEA. (E) Iso-affinity plot summarizing the kinetic constants of 27 purified nanobody candidates measured by biolayer interferometry (BLI). The *x*-axis represents the association rate (*k_on_*), and the *y*-axis represents the dissociation rate (*k_off_*). Diagonal lines indicate iso-affinity (*K_D_*) values. (F) Sodium dodecyl sulfate–polyacrylamide gel electrophoresis (SDS-PAGE) analysis of purified nanobody variants (CE8, D7, B6, and C10). The gel confirms the high purity and homogeneity of the proteins used for kinetic analysis. (G) Representative BLI sensorgrams of the parental antibody (CE8) and the top 3 affinity-matured variants (D7, B6, and C10) binding to CEA at indicated concentrations. (H) Table summarizing the detailed kinetic parameters (*k_on_*, *k_off_*, and *K_D_*) and the fold improvement in affinity for the top variants compared to the parental antibody.

To isolate variants with superior binding properties from the constructed library, we performed 3 rounds of stringent competitive biopanning against the CEA antigen (Fig. 3A). To drive the selection toward high-affinity clones, the stringency was progressively increased by reducing the antigen coating concentration in each subsequent round. Crucially, to specifically enrich for variants with higher affinity than the parental antibody, we implemented a competitive elution strategy using 15 μg/ml of CE8. This selection pressure resulted in a pronounced enrichment profile: the output-to-input phage ratio increased by several orders of magnitude from the first to the third round (Fig. 3B), indicating the successful amplification of specific binders. This enrichment was further validated by polyclonal phage ELISA, which showed a clear, progressive saturation of binding signals across the 3 rounds (Fig. 3C).

Following the third round of selection, we randomly picked 96 individual clones for monoclonal phage ELISA to assess the library’s convergence. Remarkably, 95 out of 96 clones (approximately 99%) exhibited strong binding signals (OD_450_ > 2.0) (Fig. 3D). This exceptionally high positive rate suggests that the library had successfully converged toward functional binders, eliminating nonbinding background noise. From this pool, 27 unique sequences were identified and expressed for kinetic screening. The iso-affinity plot (Fig. 3E) derived from biolayer interferometry (BLI) measurements revealed a comprehensive landscape of affinity maturation.

The high-fidelity focused library yielded an exceptional hit rate of high-performance binders. Among the 27 unique variants characterized, a total of 20 clones (74.1%) achieved subnanomolar affinities. While the 3 most potent candidates are prioritized in the main text, the detailed kinetic sensorgrams and parameters for the remaining 24 high-affinity variants are provided in Fig. S2. This broad spectrum of enhanced affinities, ranging from 44.1 pM to 7.31 nM, provides compelling empirical evidence that our physics-informed strategy of targeting thermodynamic hotspots is a highly reproducible and efficient engine for antibody maturation.

Based on the screening data, 3 top-performing candidates—D7, B6, and C10—were selected for large-scale expression and purification. Sodium dodecyl sulfate–polyacrylamide gel electrophoresis (SDS-PAGE) analysis confirmed that these variants could be purified to high homogeneity with no visible aggregation (Fig. 3F), ensuring that subsequent kinetic measurements reflected intrinsic molecular interactions rather than artifacts.

As detailed in the kinetic parameter table (Fig. 3H), the lead candidate D7 exhibited an ultrahigh affinity (*K_D_*) of 44.1 pM, representing a remarkable 306-fold improvement over the parental CE8 (*K_D_* = 13.5 nM). Similarly, clones B6 and C10 achieved affinities of 52.0 and 81.6 pM, corresponding to 260-fold and 165-fold improvements, respectively. Notably, this improvement was predominantly driven by a drastic reduction in the dissociation rate (*k_off_*), which decreased by 2 orders of magnitude from 2.12 × 10^−3^/s for CE8 to the 10^−5^/s range for the matured variants (e.g., 1.843 × 10^−5^/s for D7). This substantial decrease in *k_off_* suggests a markedly prolonged residence time on the target, providing a biophysical prerequisite for sustained tumor accumulation in vivo.

To evaluate the quantitative advantages of our physics-informed AI framework, we benchmarked its performance against both experimental and computational standards. Traditional directed evolution typically relies on massive libraries to compensate for functional hit rates that are frequently lower than 0.1% [31]. In contrast, our pipeline compressed the sequence space into a focused library of only 2.7 × 10^4^ variants, achieving an initial hit rate of 16.7% (16/96 clones) in the unselected library (Fig. 2F). This represents a >160-fold enrichment in functional sequences compared to the stochastic baseline. While recent state-of-the-art AI approaches, such as protein language models (PLMs) and structure-informed unsupervised evolution, have improved beneficial mutation rates (typically 14% to 71%) [32,33], their affinity gains are often limited, ranging from 7-fold for mature antibodies to 160-fold for precursors. Our framework’s ability to resolve subtle interfacial energetic frustrations facilitated a 306-fold affinity gain, culminating in a terminal affinity of 44.1 pM. This demonstrates superior efficiency and performance over both purely stochastic and purely generative approaches.

### Biophysical characterization and stability assessment of top mutants

**Fig. 4.**
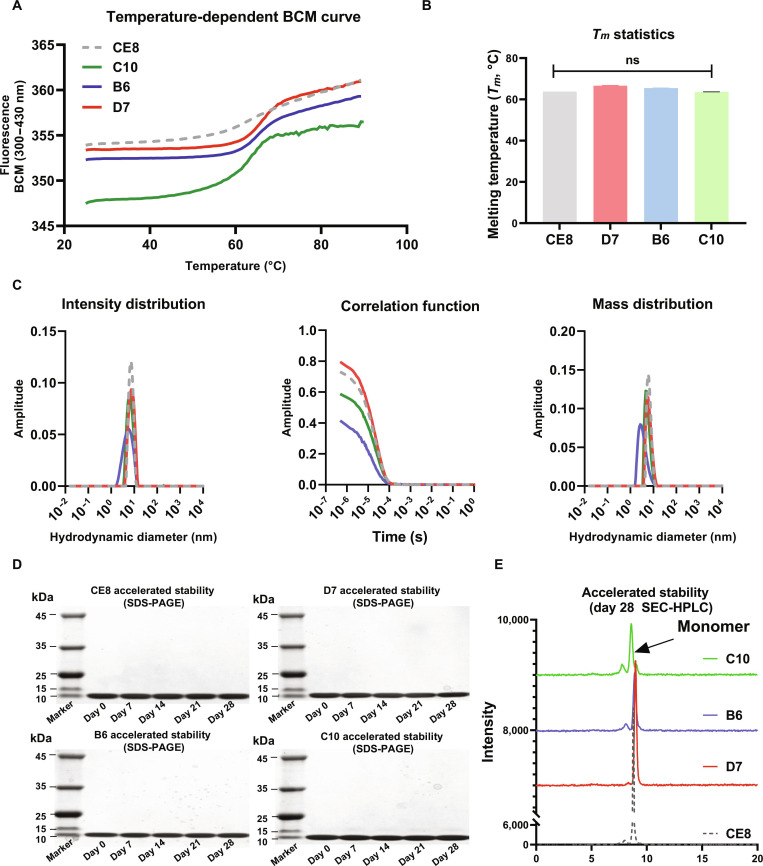
Biophysical characterization and stability profiling of nanobody variants. (A) Thermal unfolding profiles monitored by intrinsic fluorescence (barycentric mean [BCM]) over a temperature range of 20 to 90 °C. (B) Melting temperatures (*T*_m_) calculated from the unfolding curves. Data represent mean ± SD (*n* = 3). Statistical significance was assessed by one-way analysis of variance (ANOVA) (ns, not significant). (C) Dynamic light scattering (DLS) measurements showing hydrodynamic diameter based on intensity distribution (left), autocorrelation functions (middle), and mass distribution (right). (D) Sodium dodecyl sulfate–polyacrylamide gel electrophoresis (SDS-PAGE) analysis of long-term accelerated stability. Nanobodies (CE8, D7, B6, and C10) were incubated at 37 °C and sampled at weekly intervals (days 0 to 28). (E) Size-exclusion high-performance liquid chromatography (SEC-HPLC) chromatograms of the variants (C10, B6, D7, and CE8) after 28 d of incubation at 37 °C. The arrow indicates the peak corresponding to the monomeric species.

A major bottleneck in antibody engineering is the affinity–stability trade-off, where mutations that enhance binding often destabilize the immunoglobulin scaffold. We first profiled the thermal unfolding behavior by monitoring changes in intrinsic tryptophan fluorescence (barycentric mean). The temperature-dependent unfolding curves of the ultrahigh-affinity variants (D7, B6, and C10) were similar to those of the parental CE8 nanobody (Fig. 4A). Quantitative analysis revealed that all variants maintained high melting temperatures (*T*_m_ > 63 °C), with no statistically significant differences observed compared to the wild-type scaffold (Fig. 4B; *P* > 0.05). Furthermore, dynamic light scattering analysis demonstrated excellent colloidal homogeneity; the variants displayed strictly monodisperse profiles with hydrodynamic diameters in the expected range (∼10 nm), indicating the absence of preexisting aggregates (Fig. 4C).

Given the “hitchhiking and locking” mechanism intended for long-term circulation, we specifically assessed the stability of these variants under simulated physiological conditions. An accelerated stability assay was performed by incubating the nanobodies at 37 °C for up to 28 d. Remarkably, all variants showed exceptional structural integrity, as evidenced by the persistent monomeric bands at ∼15 kDa on SDS-PAGE with no detectable fragmentation or degradation products over the 4-week period (Fig. 4D). Corroborating these results, size-exclusion high-performance liquid chromatography (SEC-HPLC) profiles after 28 d of incubation at 37 °C revealed that the lead candidates maintained a predominant monomeric peak with no evidence of heat-induced aggregation (Fig. 4E). To provide a more granular validation of this conformational stability, we performed longitudinal SEC-HPLC comparisons for each individual variant (Fig. S3). The elution profiles at day 28 remained virtually indistinguishable from their respective day 0 baselines, showing near-perfect alignment with no detectable high-molecular-weight species or appreciable shifts in retention time. Collectively, these biophysical data establish that our AI-guided, structure-based design successfully defies the conventional trade-off, delivering picomolar binders that possess the robust developability profile required for complex in vivo applications.

### Rational de-immunization of the optimized nanobody scaffold

To assess the translational potential of our engineered nanobodies, we utilized the NetMHCIIpan-4.0 algorithm to predict MHC-II-restricted CD4^+^ T-cell epitopes across 10 high-frequency HLA-DRB1 alleles, namely, DRB1-0101, DRB1-0301, DRB1-0401, DRB1-0405, DRB1-0701, DRB1-0901, DRB1-1101, DRB1-1201, DRB1-1302, and DRB1-1501 (Table S4). The parental CE8 nanobody harbored a substantial immunogenic liability, containing 22 strong-binding (SB) epitopes distributed across 5 major alleles (DRB1-0101, DRB1-0401, DRB1-0405, DRB1-0701, and DRB1-0901). In contrast, the lead candidate D7 lacked SB epitopes entirely across the 10-allele panel. These data indicate that the rational mutations designed to overcome thermodynamic bottlenecks concurrently de-immunized the scaffold. Ultimately, this dual benefit of enhanced binding affinity and minimized immunogenic risk highlights D7 as an optimal candidate for clinical precision imaging.

### Engineering and biophysical characterization of bispecific nanobodies

To generate long-circulating probes for in vivo imaging, we engineered a panel of bsAbs through a modular design approach. To maintain the ultrahigh affinity of the matured nanobodies and circumvent steric hindrance, we utilized a rational semirigid linker instead of conventional flexible linkers. Specifically, the optimized structured linker (GGGGGSASTKGPSVGGGSGGS) features an ASTKGPSV motif derived from the human IgG1 CH1–CL interface, which introduces a defined structural kink. This spatial separation of the anti-CEA and anti-HSA VHH domains prevents domain entanglement and facilitates independent, noncompetitive binding to both target antigens.

**Fig. 5.**
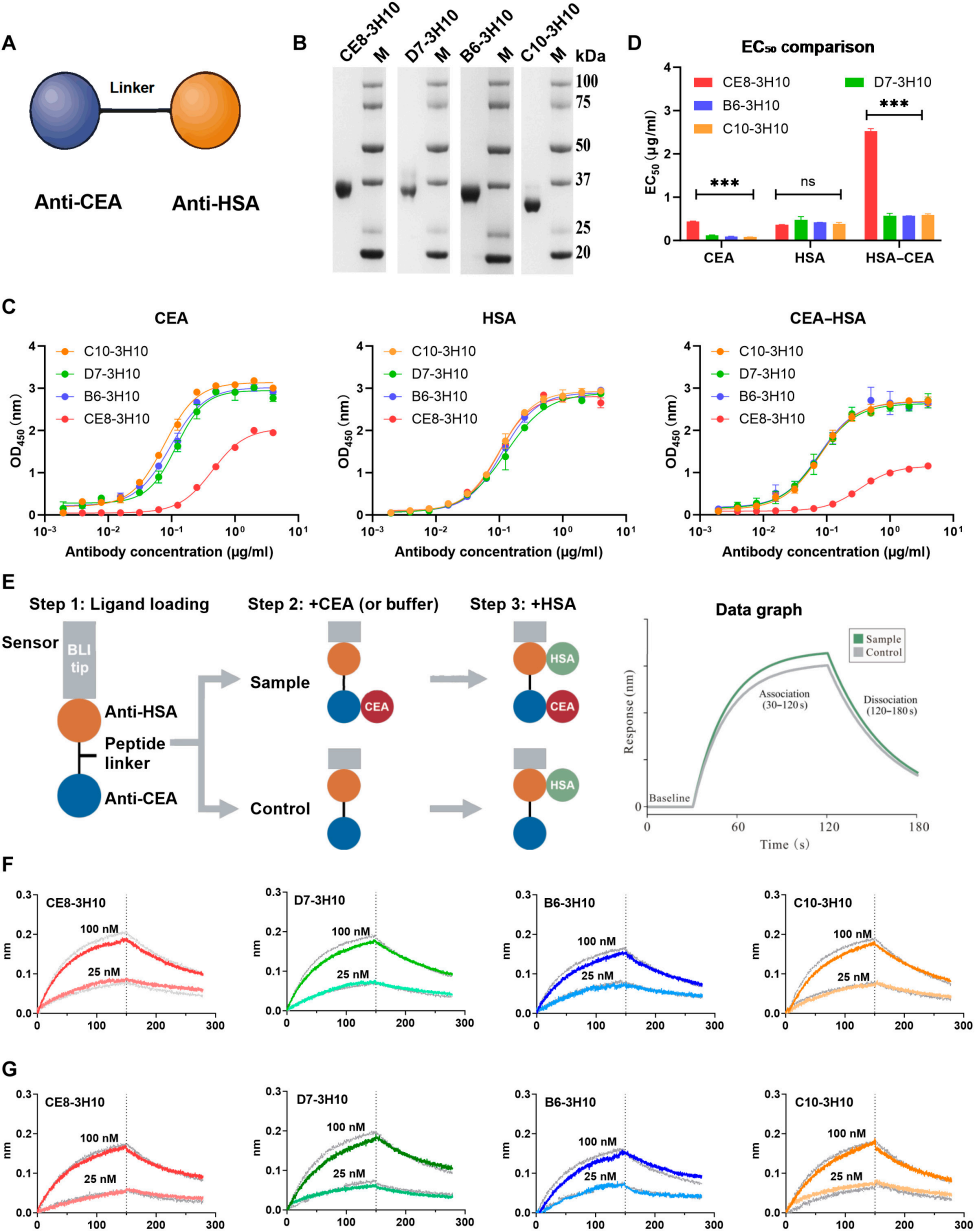
Design, purification, and functional validation of carcinoembryonic antigen (CEA)/human serum albumin (HSA)-bispecific nanobodies. (A) Schematic representation of the bispecific nanobody format, consisting of an anti-CEA VHH fused to an anti-HSA VHH via a semirigid structured linker (GGGGGSASTKGPSVGGGSGGS). (B) Sodium dodecyl sulfate–polyacrylamide gel electrophoresis (SDS-PAGE) analysis of the purified bispecific antibodies (CE8-3H10, D7-3H10, B6-3H10, and C10-3H10). M: protein marker. (C) Binding characterization by enzyme-linked immunosorbent assay (ELISA). The panels display dose–response curves for binding to CEA (left), HSA (middle), and simultaneous dual-antigen binding in a sandwich format (right). (D) Comparison of EC_50_ values derived from the ELISA data. The affinity-matured variants exhibit substantially lower EC_50_ values for CEA compared to the parental CE8-3H10, while HSA binding remains unchanged. Data are mean ± SD (*n* = 3). ***P < 0.001; ns, not significant (one-way analysis of variance [ANOVA]). (E) Schematic of the biolayer interferometry (BLI) assay designed to test for steric hindrance. The sensor tip is loaded with the bispecific antibody, followed by sequential exposure to CEA (or buffer) and then albumin (HSA/mouse serum albumin [MSA]). (F and G) BLI sensorgrams showing the binding kinetics to HSA (F) and MSA (G). Curves represent the association and dissociation phases of the antibody binding to albumin in the presence (colored lines) or absence (gray lines) of prebound CEA. The overlapping curves indicate that CEA occupancy does not sterically inhibit albumin binding. Sequential BLI binding assays confirm that CEA and HSA binding are mutually independent, with no steric interference between the 2 VHH domains.

The affinity-matured anti-CEA VHHs (D7, B6, and C10) and the parental clone (CE8) were genetically fused to an HSA-binding nanobody (3H10) via this optimized semirigid linker (Fig. 5A). The constructs were expressed in CHO-S cells and purified to homogeneity. SDS-PAGE analysis revealed a single, distinct band at approximately 35 kDa for all variants, consistent with their theoretical molecular weights (Fig. 5B). SEC-HPLC further confirmed the structural integrity of the proteins, showing a monomeric purity exceeding 90% with no observable aggregation (Fig. S4A to D).

We next characterized the antigen-binding profiles using ELISA. As shown in Fig. 5C, all bispecific constructs displayed dose-dependent binding to both CEA and HSA. Importantly, in a sandwich ELISA format, the bsAbs successfully bridged the 2 antigens, confirming dual specificity. Quantitative analysis of the EC_50_ values (Fig. 5D) demonstrated that the affinity-matured variants (D7-3H10, B6-3H10, and C10-3H10) retained markedly enhanced binding toward CEA compared to the parental CE8-3H10 (*P* < 0.001) while maintaining comparable affinity for HSA (*P* > 0.05).

To rigorously assess whether the bispecific construct introduces steric hindrance between the 2 VHH domains, we performed a sequential binding assay using BLI (Fig. 5E). We monitored the association kinetics of the bsAbs to albumin (HSA or mouse serum albumin [MSA]) in the presence and absence of saturating concentrations of CEA. The resulting sensorgrams revealed that pre-complexation with CEA did not alter the binding kinetics for either HSA (Fig. 5F) or MSA (Fig. 5G). Furthermore, the ratio of binding signals (signal with CEA/signal without CEA) remained close to unity (range: 0.90 to 1.22) across all tested concentrations (Fig. S4E and F). This lack of steric hindrance confirms that the 2 VHH binding sites operate independently, ensuring that albumin-mediated half-life extension does not compromise tumor recognition.

### Affinity-matured bispecific nanobodies demonstrate superior tumor accumulation and deep tissue penetration

To validate the translational potential of our thermodynamically driven design, we evaluated the in vivo biodistribution and tumor-targeting efficacy of the bispecific nanobodies in LS174T tumor-bearing xenograft mice. Longitudinal near-infrared fluorescence imaging was performed over a 168-h timeframe to compare the parental construct (CE8-3H10), the high-affinity variants (D7-3H10, B6-3H10, and C10-3H10), and the nontargeting control (3H10). To ensure sufficient statistical power and adequately account for inherent biological variability, we utilized an expanded cohort of *n* = 9 biologically independent mice per group.

**Fig. 6.**
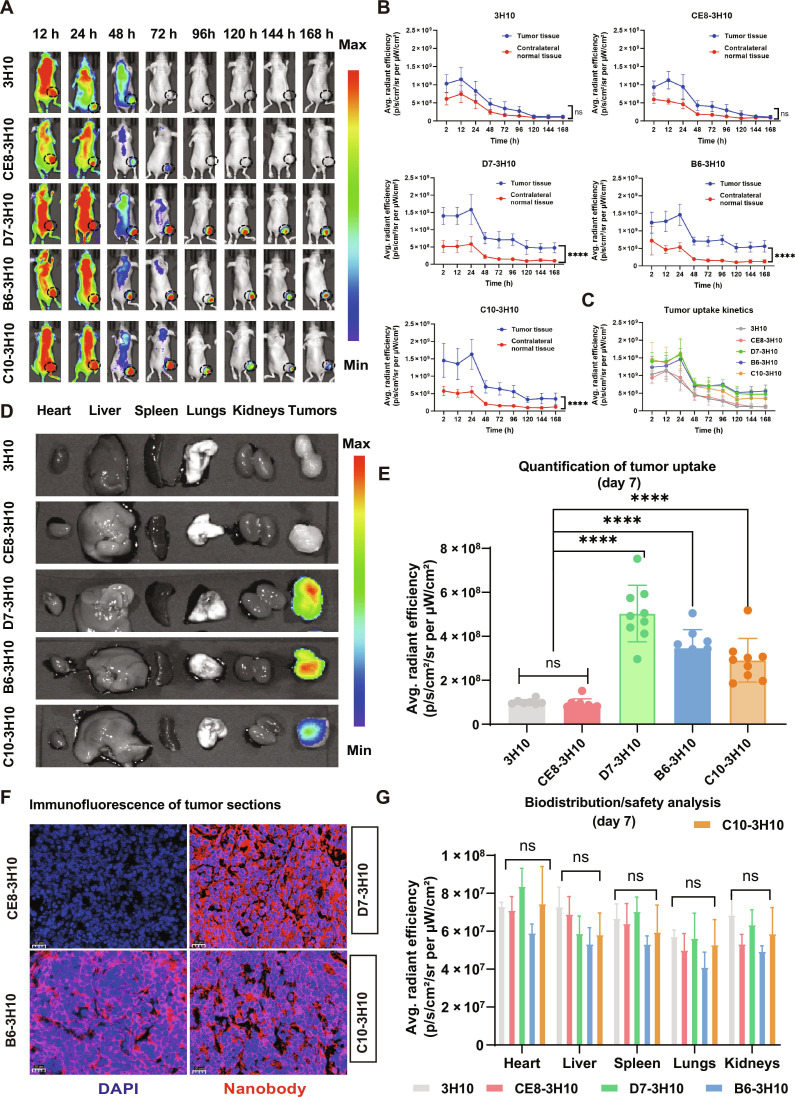
Affinity maturation confers superior tumor accumulation and sustained retention in vivo. (A) Representative longitudinal in vivo near-infrared (NIR) fluorescence images of LS174T tumor-bearing mice following intravenous injection of cyanine5 (Cy5)-labeled bispecific nanobodies. Images were acquired at the indicated time points over a 168-h period. Black dashed circles delineate tumor locations. The color scale reflects radiant efficiency. (B) Spatiotemporal distribution and contrast analysis. Individual kinetic plots for each experimental cohort (3H10, CE8-3H10, D7-3H10, B6-3H10, and C10-3H10) comparing the average radiant efficiency in tumor tissue (blue) versus contralateral normal tissue (red) over 168 h. (C) Comparative tumor-specific accumulation kinetics across all groups, based on average radiant efficiency over time. (D) Ex vivo fluorescence imaging of excised tumors and major organs collected at the experimental endpoint (day 7). (E) Quantitative analysis of the average radiant efficiency in excised tumors at day 7. (F) Representative immunofluorescence images of frozen tumor sections illustrating the microscopic intratumoral distribution of the nanobodies at day 7 post-intravenous administration. Nanobodies were labeled with Cy5 (red), and nuclei were counterstained with 4′,6-diamidino-2-phenylindole (DAPI; blue). Scale bars: 20 μm. (G) Ex vivo biodistribution analysis quantifying the average radiant efficiency in major organs at day 7, demonstrating no substantial differences in nonspecific off-target uptake among the groups. Data in (B), (C), (E), and (G) are presented as mean ± SD (*n* = 9 biologically independent animals per group). ****P<0.0001; ns, not significant.

Spatiotemporal fluorescence analysis revealed distinct in vivo PK profiles among the experimental groups (Fig. 6A). The nontargeting control 3H10 and the parental CE8-3H10 were rapidly cleared from systemic circulation, with negligible tumor fluorescence detectable by 48 h. In contrast, the affinity-matured variants displayed a pronounced “lock-and-hold” tumor retention phenotype. Quantitative spatiotemporal analysis of tumor fluorescence dynamics corroborated these phenotypic observations (Fig. 6B), with all high-affinity variants maintaining statistically significantly higher tumor signal intensities than the parental clone (P<0.0001).

An assessment of tumor uptake kinetics across the expanded cohort further highlighted the PK superiority of the optimized variants (Fig. 6C). Throughout the 168-h observation window, all 3 matured clones exhibited markedly higher absolute tumor accumulation compared to the CE8-3H10 and 3H10 controls. Notably, while C10-3H10 reached its maximum tumor signal at 24 h before undergoing subsequent signal attenuation, D7-3H10 and B6-3H10 exhibited superior lock-and-hold characteristics. Following the initial tissue distribution phase, the tumor fluorescence signals for D7 and B6 plateaued and remained highly stable from 48 to 168 h. At the experimental endpoint, D7-3H10 and B6-3H10 demonstrated highly overlapping, sustained tumor retention, substantially outperforming C10-3H10. These kinetic profiles suggest that while rapid initial tumor uptake is beneficial, the ultraslow dissociation kinetics characteristic of D7 and B6 serve as the critical determinant for long-term, high-contrast tumor imaging.

To definitively confirm this lock-and-hold retention effect, mice were euthanized at 168 h (day 7) for ex vivo near-infrared fluorescence imaging. Ex vivo scanning of excised organs (Fig. 6D) revealed intense and highly specific tumor accumulation for the high-affinity variants (D7-3H10, B6-3H10, and C10-3H10). In contrast, tumor tissues from the CE8-3H10 and nontargeting 3H10 cohorts displayed no detectable red fluorescence. Quantitative analysis of the average radiant efficiency validated these macroscopic observations (Fig. 6E). The lead candidate D7-3H10 exhibited profoundly enhanced tumor retention relative to the parental clone CE8-3H10 (P<0.0001). Consistent with the in vivo imaging, all 3 affinity-matured variants yielded markedly stronger terminal tumor signals, whereas no statistical difference was observed between the parental CE8-3H10 and the nontargeting control 3H10 (ns). Crucially, biodistribution analysis confirmed the absence of nonspecific accumulation in major metabolic organs, including the heart, liver, spleen, lungs, kidneys, and additional tissues including skin, muscle, brain, stomach, and intestine across all groups (Fig. 6G and Fig. S5). This endpoint evaluation compellingly demonstrates that the optimized binding energetics confer stable, tumor-specific in vivo accumulation without exacerbating off-target retention.

Beyond macroscopic biodistribution, we further investigated the microscopic intratumoral penetration of the nanobodies via immunofluorescence staining (Fig. 6F). At day 7 post-administration, no red fluorescent signal was detected in tumor sections from the CE8-3H10 group, consistent with its poor intratumoral retention. In contrast, the high-affinity variants (D7-3H10, B6-3H10, and C10-3H10) exhibited extensive, deep, and relatively uniform intratumoral distribution. The fluorescent signals were not restricted to perivascular niches but permeated extensively throughout the tumor parenchyma, clearly delineating cellular membranes. This finding indicates that the optimized biophysical properties of these variants facilitate efficient extravasation and penetration into dense tumor stroma, effectively overcoming the classical BSB that frequently limits the efficacy of conventional high-affinity macromolecular targeting agents.

## Discussion

Affinity maturation of nanobodies to the picomolar regime, without compromising structural stability, remains a major challenge [11]. While recent advances in generative AI—such as ProteinMPNN and RFdiffusion—have revolutionized macromolecular design by enabling the exploration of vast sequence spaces, their application is currently limited by the scarcity of high-resolution structural data linking sequence to dynamic function [34–37]. Consequently, while AI excels at capturing geometric patterns and accurately mapping static protein binding interfaces (e.g., utilizing advanced biospatial convolutions like SpatConv) [38], it often lacks the precision to predict fine-grained dynamic thermodynamic properties like binding free energy (ΔG) and kinetic off-rates. Purely generative outputs, therefore, often require rigorous refinement to meet the energetic and developability criteria necessary for clinical translation [36,37,39].

To address this, we established a synergistic physics-informed AI-driven framework, which perfectly exemplifies the emerging consensus that next-generation drug development relies on the tight iteration between generative AI and physically grounded computation [40]. In this paradigm, AI serves as the creative engine to rapidly propose diverse structural candidates, while MD and MM/GBSA calculations provide the physical ground truth by explicitly modeling atomic-level interactions [21,27,41]. Unlike static geometric predictions, this physics-based integration captures the dynamic conformational landscapes and solvent effects that govern binding affinity [42]. By using AI to generate high-fidelity structural primitives and physics to enforce thermodynamic constraints, we shift the workflow from the “blind screening” of massive libraries to the targeted interrogation of variants that are both geometrically sound and energetically favorable [37,39,43,44].

Crucially, this targeted strategy is both highly resource efficient and computationally reproducible (detailed in Table S3). On a standard high-performance computing (HPC) setup, the entire pipeline—encompassing AlphaFold 3 modeling, 100-ns MD simulations, and vd-MM/GBSA thermodynamic decomposition—requires only 68 to 131 h. By focusing mutagenesis on just 8 rationally identified interface hotspots rather than relying on full-library randomization, we achieved an initial (unscreened) hit rate of 16.7% and a 99% binding positivity rate after 3 rounds of panning. This efficiency is highly competitive with recent state-of-the-art AI-driven maturation approaches, such as general PLMs, which typically report beneficial mutation rates of 14% to 71% and affinity improvements ranging from 7-fold (for mature antibodies) to 160-fold (for germline precursors) [32,33]. In contrast, our hybrid pipeline successfully yielded variants with a terminal affinity of $K_{D}=44.1\;\mathrm{pM}$, representing a remarkable 306-fold improvement.

The efficacy of this hybrid approach is evidenced by the resolution of specific interfacial defects often overlooked by geometric methods. While AlphaFold 3 facilitated initial backbone prediction, subsequent “interfacial microenvironment optimization”—guided by vd-MM/GBSA calculations—identified critical energetic frustrations. Affinity gains were achieved not by altering the global fold but by refining local thermodynamics: optimizing electrostatic networks (e.g., D108Y), minimizing hydrophobic desolvation penalties (e.g., F32Q), and improving van der Waals packing (e.g., S28T). Notably, thermal shift assays confirmed that the *T*_m_ values of matured variants were comparable to that of the wild type, indicating that our protocol successfully decoupled affinity improvement from the destabilization frequently associated with error-prone PCR [9,16,17].

Kinetic characterization revealed that the ~306-fold improvement in affinity was primarily driven by a marked reduction in the dissociation rate constant (*k_off_*). This extended residence time is critical for imaging probes, where signal retention is paramount. To translate this biophysical advantage into in vivo performance, we fused the high-affinity binder with an anti-HSA nanobody [45]. This design mitigates the rapid renal clearance typical of small nanobodies, extending plasma half-life and maintaining a systemic concentration gradient that drives tumor uptake [46–48]. While traditional half-life extension strategies often rely on Fc-fusions—which frequently require additional engineering, such as Fc-silencing mutations, to abrogate off-target toxicities [49]—our use of a compact anti-HSA nanobody module effectively circumvents these liabilities while preserving the diminutive hydrodynamic radius crucial for deep tumor penetration.

In vivo imaging validated this dual-targeting strategy, demonstrating a “lock-and-hold” phenotype characterized by high tumor accumulation and a favorable tumor-to-normal ratio. In stark contrast to conventional monomeric nanobodies, which typically visualize tumors rapidly but are cleared within hours [50], our bispecific probe achieved deep penetration and sustained retention exceeding 168 h. These findings also challenge the BSB hypothesis, which predicts that ultrahigh-affinity agents ($K_{D}<1\;\mathrm{nM}$) are sequestered at the tumor periphery [4–6]. Contrary to this, immunofluorescence analysis showed homogeneous intratumoral distribution. We propose that this deep penetration is facilitated by the nanobody’s small hydrodynamic radius (~2.5 nm) and high diffusion coefficient, combined with the extended temporal window provided by the HSA binder. Thus, the combination of picomolar affinity and extended circulation synergistically enhances both signal intensity and spatial resolution [45,51].

From a clinical translation perspective, successful biophysical optimization must be reconciled with biological constraints, most notably immunogenicity [52]. While nanobodies are generally regarded as low-risk scaffolds due to their high sequence identity with the human VH3 germline [53], the introduction of multiple affinity-maturing mutations presents a theoretical risk of creating neo-epitopes. To address this, our “interfacial microenvironment optimization” strategy was deliberately designed to inherently avoid introducing known immunogenic hotspots by prioritizing a minimalist set of mutations at the binding interface. Quantitative in silico profiling via NetMHCIIpan-4.0 [54] across 10 major HLA-DRB1 alleles confirmed the success of this approach: the parental clone (CE8) contained 22 SB CD4^+^ T-cell epitopes, whereas our lead optimized variant (D7) exhibited zero SB epitopes across all tested alleles (Table S4). This concomitant achievement of a 306-fold affinity enhancement and a comprehensive “de-immunization” of the protein scaffold strongly supports the translational potential of our design strategy.

Beyond immunogenicity, the transposability of this pipeline depends on the antigen’s conformational landscape. Our “identify–decompose–optimize” logic is fundamentally target agnostic; however, its practical performance is influenced by the antigen’s structural heterogeneity. The CEA-D1 domain utilized here represents a relatively rigid IgV-like fold [55], which is conducive to MD-based refinement and AlphaFold 3 -based structural priors. For more structurally dynamic targets, such as G-protein-coupled receptors [56] or flexible viral glycoproteins [57], the pipeline would likely benefit from enhanced ensemble sampling to accurately capture the dynamic energy landscape of the interface [58].

It is important to emphasize that our physics-constrained approach is not mutually exclusive with emerging pure AI-driven generative models; rather, they represent a highly complementary paradigm. Recent breakthroughs in deep learning have introduced powerful tools that generally fall into 2 distinct categories: de novo binder generation and AI-guided affinity maturation. On one hand, diffusion models (e.g., RFdiffusion and BindCraft) have revolutionized de novo design by rapidly exploring vast sequence spaces to generate novel binding interfaces [31,35]. On the other hand, AI-guided maturation methods, such as PLMs and antibody-specific diffusion models (e.g., AbDiffuser), excel at navigating evolutionary sequence landscapes to identify beneficial mutations with remarkably high success rates [32,37]. While these AI-centric methods represent a profound paradigm shift [21], their reliance on static structural priors and training data distributions means that they can sometimes lack the fine-grained physical resolution required to accurately predict dynamic thermodynamic trade-offs or resolve subtle energetic frustrations in novel complexes. Here, our physics- and chemistry-constrained framework provides a critical auxiliary function: it serves as an effective downstream optimization module that offers precise structural computation and rigorous thermodynamic validation.

Ultimately, this work demonstrates that integrating AI-driven structural predictions with high-fidelity physical simulations provides a reliable blueprint for accelerating the development of next-generation biologics. Moving forward, the most effective affinity maturation pipelines will likely be those that couple the expansive, high-throughput search capabilities of deep learning with the precise energetic filtering of MD and MM/GBSA. By functioning as a modular “identify–decompose–optimize” cascade, our workflow supports the seamless integration of continually advancing AI technologies, creating a synergistic loop where AI proposes expansive solutions and physics ensures their thermodynamic viability for clinical translation.

## Materials and Methods

### Reagents, cell lines, and animals

Recombinant human CEA (Cat. 11077-H08H) and CEA-Fc fusion protein (Cat. 11077-H02H) were purchased from Sino Biological (Beijing, China). HSA (Cat. A8230) and MSA (Cat. SP060) were obtained from Solarbio (Beijing, China).

Antibodies and labeling reagents included: horseradish peroxidase (HRP)-conjugated anti-M13 phage antibody (Sino Biological, Cat. 11973-MM05T), anti-VHH Cocktail [HRP] (GenScript, Cat. A02016), HRP-conjugated goat anti-human IgG Fc secondary antibody (F(ab′)_2_ fragment; GenScript, Cat. A10254), and sulfo-cyanine5 (sulfo-Cy5) *N*-hydroxysuccinimide ester (Qiyue Biology, Cat. R-H-5029).

Molecular biology reagents included 2× PrimeSTAR Max Premix (Takara Bio, Cat. R045A), DL2000 DNA Marker (Takara, Cat. 3427Q), 6× DNA Loading Buffer (Takara, Cat. 9156), SfiI restriction endonuclease (New England Biolabs, Cat. R0123S), and T4 DNA Ligase (New England Biolabs, Cat. M0202L).

The phagemid vector pComb3XTT and TG1 electrocompetent cells (Lucigen, Cat. 60502-2) were used for library construction. All primers were synthesized and HPLC-purified by GenScript (Nanjing, China). All other analytical-grade chemical reagents were purchased from Sigma-Aldrich, NEB, or Sinopharm unless otherwise stated.

The human colorectal cancer cell line LS174T (high CEA expression) was obtained from the American Type Culture Collection. CHO-S cells and BL21 (DE3) strains were maintained in our laboratory. Female BALB/c nude mice (6 to 8 weeks, 18 to 22 g) were purchased from Beijing Vital River Laboratory Animal Technology Co., Ltd. All animal procedures were conducted in strict accordance with the Guidelines for the Care and Use of Laboratory Animals. The study protocol was reviewed and approved by the Institutional Animal Care and Use Committee under the approval number DWZX-IACUC-2025-P559.

### Epitope mapping via flow cytometry

To define the binding region of the parental nanobody, the full-length extracellular domain of CEA and its C-terminal truncated variants (CEA-D1 to CEA-D1-7) were cloned into the pcDNA3.4 vector. CHO-FLP-IN cells were transfected using Lipofectamine 3000 to generate stable cell lines. Cells were harvested, washed with phosphate-buffered saline (PBS) containing 1% bovine serum albumin, and incubated with the parental CE8 nanobody (10 μg/ml) for 30 min at 4 °C. Binding was detected using an allophycocyanin-conjugated anti-His tag antibody on a BD FACSCelesta flow cytometer. Data were analyzed using the FlowJo software to identify the specific domain essential for CE8 binding.

### Structure-based library design and hotspot identification

To bridge the gap between static structural predictions and dynamic binding realities, we established a physics-informed rational design workflow integrating AlphaFold 3 modeling, consensus docking, and rigorous thermodynamic simulation.

#### High-precision structural modeling and docking

The amino acid sequences of the antigen epitope and the parental nanobody were processed using AlphaFold 3. To ensure unbiased modeling, homologous templates were strictly excluded. Model quality was assessed based on predicted template modeling scores and predicted aligned error matrices.

The highest-confidence monomer models were subjected to protein–protein docking using the HADDOCK2.4 docking platform. A global search was performed to generate potential binding poses. To identify the optimal binding mode, these poses were rescored using a weighted consensus scoring function that integrates AMBER, AMOEBA, and DFIRE force fields. The conformation achieving the highest composite score was selected as the starting complex for subsequent maturation.

#### Dynamic stability verification and MD simulation

To assess the thermodynamic stability of the predicted interface, the complex was subjected to 100 ns of classical MD simulation (GROMACS 2024.2). Trajectory analysis parameters included root-mean-square deviation, radius of gyration (*R_g_*), solvent-accessible surface area, and per-residue root-mean-square fluctuation. The Gibbs free energy landscape was constructed by projecting the trajectory onto the principal components of the system to visualize the energy basins of the conformational space.

#### Thermodynamic decomposition and hotspot identification

The equilibrated structure was subjected to granular thermodynamic profiling using the vd-MM/GBSA decomposition method. The immediate interaction shell was defined as residues located within 6 Å of the antigen interface. The total binding free energy was dissected into individual residue contributions. Residues were flagged as thermodynamic hotspots for engineering if they met 2 criteria:

• spatial proximity: located within the defined interaction shell
• energetic liability: exhibited a binding free energy contribution > −3.0 kcal/mol, serving as a threshold to identify residues making suboptimal contributions to the binding enthalpy

#### Interfacial microenvironment optimization and in silico screening

To resolve the identified thermodynamic bottlenecks, a rational design strategy termed interfacial microenvironment optimization was applied. Mutations were computationally designed based on 4 specific physical mechanisms:

• electrostatic and hydrogen-bond enhancement: introducing polar residues to reduce steric hindrance and establish directional hydrogen bonds
• solvation effect optimization: substituting exposed hydrophobic residues with polar groups to minimize desolvation penalties
• charge balance and interaction upgrades: neutralizing electrostatic repulsion and introducing stabilizing aromatic π–π stacking
• shape complementarity optimization: modifying side-chain volume to fill interfacial packing defects and enhance van der Waals contacts

In silico saturation mutagenesis was performed at the identified hotspot positions. The relative binding free energy (Δ*G*_bind_) was calculated for each variant relative to the wild-type complex. Mutations predicting a binding free energy lower than that of the wild type were retained as affinity-enhancing candidates.

#### Combinatorial focused library construction

To explore epistatic interactions, the high-scoring variants identified in the in silico screen were integrated into a combinatorial focused library. The selected mutations were mathematically permuted across the targeted positions within the complementarity-determining regions (CDRs) to generate a defined sequence space for experimental validation.

#### Computational implementation and resource allocation

All computational simulations were performed on an HPC cluster equipped with NVIDIA A100 (80 GB) graphics processing units (GPUs) and Intel Xeon Platinum central processing units. The total computational footprint for the anti-CEA affinity maturation was approximately 68 to 131 GPU-hours, depending on the sampling depth of MD trajectories. Structural modeling was executed via a local Docker implementation of AlphaFold 3. MD simulations were performed using GROMACS 2024.2 with the AMBER99SB-ILDN force field. The pipeline is designed to be hardware agnostic and highly scalable, requiring only standard HPC resources common in modern biopharmaceutical research. The detailed time allocation for each stage is provided in Table S3.

### Library construction and phage display selection

#### Library construction

The focused nanobody library was constructed via a multistep overlap extension PCR strategy.

##### Primary PCR for fragment generation

For targeted mutagenesis sites utilizing a split-synthesis strategy, the corresponding degenerate primers were premixed at specific molar ratios proportional to their codon degeneracies (as detailed in Table S2) to ensure an equimolar representation of the encoded amino acids. Fragments were amplified in 50-μl reactions containing 50 ng of the template, 1 μl of each premixed forward and reverse primer pool (10 μM), and 25 μl of 2× PrimeSTAR Max Premix. Thermal cycling was performed as follows: 98 °C for 30 s; 35 cycles of 98 °C for 15 s, 55 °C for 30 s, and 72 °C for 30 s; followed by a final extension at 72 °C for 120 s.

##### Electrophoresis and purification

The PCR products were resolved on a 1.0% (w/v) agarose gel at a constant 150 V for 30 min using a Bio-Rad Electrophoresis System (USA; Cat. No. 1645050). Gels were visualized using a JY04S-3C Gel Imaging Analysis System (Junyi Dongfang, China). Target bands were excised and purified using the QIAquick Gel Extraction Kit according to the manufacturer’s instructions.

##### Overlap PCR assembly

The purified upstream and downstream fragments (35 ng each) were assembled in a 50-μl reaction containing 25 μl of 2× PrimeSTAR Max Premix. To ensure precise overlap annealing and extension, an initial 8 cycles were performed without flanking primers (98 °C for 10 s, 60 °C for 30 s, and 72 °C for 30 s). Subsequently, 1 μl of each flanking primer (10 μM) was added to the reaction mixture, followed by an additional 35 cycles of amplification using the same thermal parameters.

#### Cloning and library transformation

The assembled VHH library and the pComb3XTT phagemid vector were double-digested with SfiI in CutSmart Buffer at 37 °C for 8 h. The digested products were gel-purified and ligated at a 1:3 vector-to-insert molar ratio using T4 DNA ligase at 16 °C for 14 h. The ligated products were then purified and transformed into competent *E. coli* TG1 cells via electroporation. Serial dilutions of the transformants were plated on 2× YT agar plates supplemented with ampicillin and glucose (2YT-Amp-Glu) to determine the library size, while the remaining transformants were cultured for subsequent phage display applications.

#### Biopanning

Three rounds of selection were performed against immobilized CEA. To drive affinity maturation, the antigen concentration was progressively reduced (10, 2, and 0.5 μg/ml) and washing stringency (time and frequency) was increased across rounds. Individual clones from the output were screened by phage ELISA, and positive hits were sequenced.

### Protein expression and characterization

The recombinant plasmids were transformed into *E. coli* competent cells. Cells were cultured in 2× YT medium supplemented with appropriate antibiotics at 37 °C until OD_600_ reached ~0.7. Protein expression was then induced with 1 mM isopropyl β-d-1-thiogalactopyranoside at 16 °C for 8 to 12 h. Harvested cells were resuspended in PBS containing 1 mM phenylmethylsulfonyl fluoride (YEASEN, China) and completely lysed by sonication on ice. After clarification by centrifugation (6,000 rpm, 10 min, 4 °C) and filtration, the supernatant was loaded onto a 1 ml Ni-NTA 6FF column (BBI, USA). Target proteins were eluted using a step gradient of imidazole (10 to 500 mM), and the purity of the eluates was verified by SDS-PAGE. Finally, the purified fractions were concentrated using polyethylene glycol and dialyzed thoroughly against PBS at 4 °C. Binding kinetics (*k_on_*, *k_off_*, and *K_D_*) were determined using an Octet BLI system. Melting temperatures (*T*_m_) were measured using NanoDSF. Long-term stability was assessed by monitoring monomer content via SEC-HPLC after incubation at 37 °C.

### Engineering and validation of bispecific probes

The affinity-matured anti-CEA nanobody was fused to an anti-HSA nanobody (clone 3H10) via a structured linker (GGGGGSASTKGPSVGGGSGGS). Specifically, the ASTKGPSV motif was incorporated to introduce a rigid structural kink, thereby minimizing steric hindrance between the 2 domains. Following expression in CHO-S cells and subsequent purification, the bsAbs were systematically validated for their functional integrity. First, an indirect ELISA was performed to confirm the individual binding capabilities to both CEA and HSA. Next, a bridging ELISA was conducted to demonstrate simultaneous dual targeting; in this assay, the bsAb was captured by immobilized HSA and subsequently detected using soluble CEA-Fc. Finally, a tandem BLI assay—involving the sequential loading of HSA and CEA onto the biosensor—was employed to verify the independent, noncompetitive binding of the 2 domains.

### In vivo and ex vivo fluorescence imaging

LS174T xenografts were established in BALB/c nude mice. Upon reaching a tumor volume of 60 to 150 mm^3^, mice (*n* = 9/group) received an intravenous injection of Cy5-labeled bsAbs (10 mg/kg; dye-to-protein ratio ≈ 4:1). In vivo fluorescence imaging was conducted using an IVIS Spectrum system at defined intervals (from 2 h to day 7). To evaluate the tumor-targeting efficacy, the fluorescence intensities of the tumor tissues and the contralateral normal tissues were longitudinally quantified using the Living Image software. At the experimental endpoint (day 7), the mice were euthanized via an overdose of anesthesia. The tumors, major organs (heart, liver, spleen, lungs, and kidneys), and specific tissues (skin, muscle, brain, and gastrointestinal tract) were harvested for ex vivo imaging and biodistribution analysis. Tumor penetration was further analyzed by fluorescence microscopy of frozen tissue sections counterstained with 4′,6-diamidino-2-phenylindole.

### Statistical analysis

All quantitative data are presented as mean ± standard deviation. Statistical analyses were conducted using GraphPad Prism 9.0. For the longitudinal biodistribution and tumor targeting data, 2-way analysis of variance (ANOVA) followed by Tukey’s multiple comparisons test was used to compare signals between tumor and normal tissues across all time points. For comparisons between 2 independent groups, a 2-tailed unpaired Student *t* test was applied. For comparisons of 3 or more independent groups, one-way ANOVA with Tukey’s post hoc test was performed. A *P* value <0.05 was considered statistically significant. *P < 0.05, **P < 0.01, ***P < 0.001, and ****P < 0.0001; *ns*, not significant.

## Data Availability

The data are available within the article and in the Supplementary Materials.
