# Supplementary material for: Physics-Informed Artificial Intelligence Design of Picomolar Nanobodies Enables Deep Tumor Penetration and High-Contrast Imaging
Source: Research (Wash D C). 2026 Jun 24;9:1325. doi: 10.34133/research.1325 (PMC13291490; doi:10.34133/research.1325)
Supplement: Supplementary 1 — Figs. S1 to S6 Tables S1 to S4 [file research.1325.f1.pdf]

Title

Physics-Informed AI Design of Picomolar Nanobodies Enables Deep Tumor Penetration and High-Contrast Imaging

Authors

Ning Shi[1,2,3†]; Caiping Ren[1,3,4*]; Liang Zhang[2]; Lei Wang[1,3,4]; Xuechen Yang[2]; Xiaobo Li[2,5]; Yangyihua Zhou[2]; Jie Wang[2]; Pinnan Zhao[2]; Chaoyan Yao[1,2,3]; Yaowei Ma[2]; Juan Tian[2,5]; Qianping Huang[1,2]; Can Xu[2]; Xiaonan Kuang[2,5]; Weidong Liu[1,3,4]; Xingjun Jiang[1,4]; Jun Ye[6*]; Xiang Gao[2*]; Longlong Luo[2*]

Affiliations

[1] Department of Neurosurgery, Xiangya Hospital, Xiangya School of Basic Medical Science, Central South University, Changsha, Hunan 410008, China.

[2] Academy of Military Medical Sciences, Beijing 100850, China.

[3] NHC Key Laboratory of Carcinogenesis, The Key Laboratory of Carcinogenesis and Cancer Invasion of the Chinese Ministry of Education, Cancer Research Institute, Xiangya School of Basic Medical Science, Central South University, Changsha, Hunan 410078, China.

[4] National Clinical Research Center for Geriatric Disease, Xiangya Hospital, Central South University, Changsha, Hunan 410008, China.

[5] Hunan Normal University School of Medicine, Changsha, Hunan 410081, China.

[6] Beijing Key Laboratory of Key Technologies for Natural Drug Delivery and Novel Formulations, Institute of Materia Medica, Chinese Academy of Medical Sciences & Peking Union Medical College, Beijing 100050, China.

***Corresponding Author：**

Caiping Ren, E-mail: rencaiping@csu.edu.cn

Jun Ye, E-mail: yelinghao@imm.ac.cn

Xiang Gao, E-mail: gaoxiang609@163.com

Longlong Luo, E-mail: luolong_long@126.com

SUPPLEMENTARY MATERIALS

Figs. S1 to S6

Tables S1 to S4

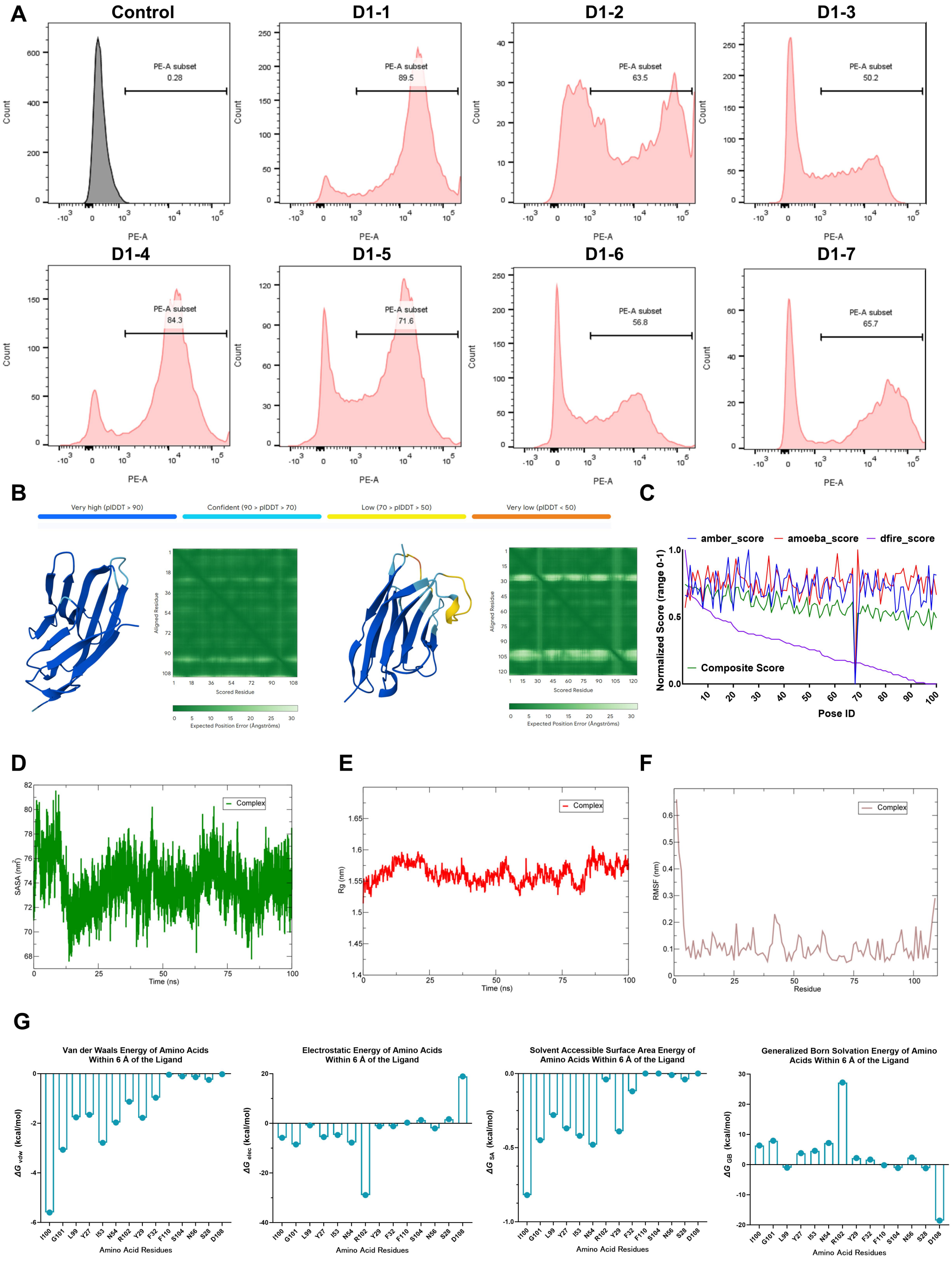

Supplementary Figure 1. Structural quality assessment and molecular dynamics validation details.

(A) Representative raw flow cytometry histograms for CEA truncation variants (D1-1 through D1-7), supporting the domain mapping results in Figure 1A. (B) AlphaFold 3 model quality assessment. Left: Structural models colored by pLDDT confidence scores (blue indicates high confidence). Right: Predicted Aligned Error (PAE) matrices indicating rigid domain packing. (C) Normalized scoring profiles of the top 100 docking poses. The

"Composite Score" (green line) represents the weighted consensus of AMBER, AMOEBA, and DFIRE force fields, identifying Pose 1 as the globally optimal binding conformation. (D–F) Time-dependent analysis of MD stability metrics over the 100 ns simulation trajectory: (D) Solvent Accessible Surface Area (SASA), (E) Radius of gyration (Rg), and (F) per-residue Root-Mean-Square Fluctuation (RMSF). (G) Component-wise decomposition of binding free energy for interfacial residues. Terms include Van der Waals (VDW), Electrostatic (Elec), non-polar solvation (SA), and polar solvation (GB).

A

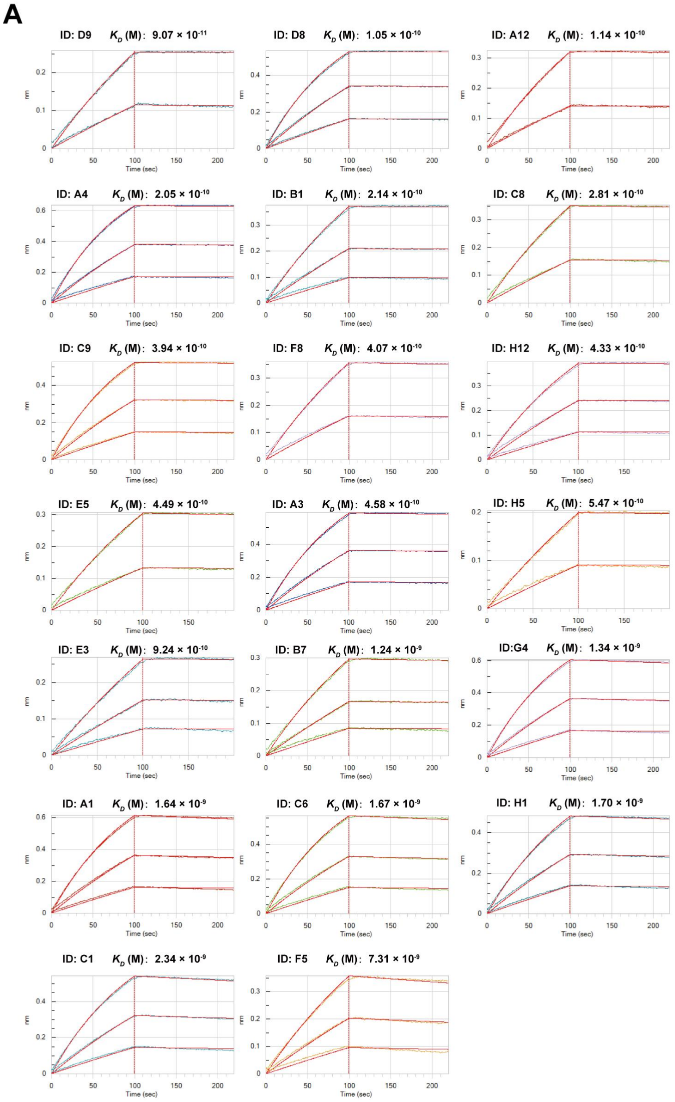

B

Clone	K_{on} (1/M s)	K_{off} (1/s)	K_D (M)
D9	2.626×10^{5}	2.382×10^{-5}	9.07×10^{-11}
D8	2.215×10^{5}	2.318×10^{-5}	1.05×10^{-10}
A12	3.766×10^{5}	4.279×10^{-5}	1.14×10^{-10}
C11	2.626×10^{5}	3.663×10^{-5}	1.39×10^{-10}
C5	2.478×10^{5}	3.762×10^{-5}	1.52×10^{-10}
A4	2.415×10^{5}	4.961×10^{-5}	2.05×10^{-10}
B1	1.203×10^{5}	2.576×10^{-5}	2.14×10^{-10}
C8	3.277×10^{5}	9.224×10^{-5}	2.81×10^{-10}
D1	2.196×10^{5}	6.526×10^{-5}	2.97×10^{-10}
G10	3.653×10^{5}	1.190×10^{-4}	3.26×10^{-10}
C9	2.082×10^{5}	8.212×10^{-5}	3.94×10^{-10}
F8	3.281×10^{5}	1.334×10^{-4}	4.07×10^{-10}
H12	1.524×10^{5}	6.597×10^{-5}	4.33×10^{-10}
E5	3.168×10^{5}	1.421×10^{-4}	4.49×10^{-10}
A3	2.038×10^{5}	9.341×10^{-5}	4.58×10^{-10}
H5	2.159×10^{5}	1.182×10^{-4}	5.47×10^{-10}
E3	6.986×10^{4}	6.454×10^{-5}	9.24×10^{-10}
B7	1.087×10^{5}	1.349×10^{-4}	1.24×10^{-9}
G4	2.069×10^{5}	2.768×10^{-4}	1.34×10^{-9}
A1	1.940×10^{5}	3.187×10^{-4}	1.64×10^{-9}
C6	2.051×10^{5}	3.430×10^{-4}	1.67×10^{-9}
H1	1.610×10^{5}	2.735×10^{-4}	1.70×10^{-9}
C1	1.845×10^{5}	4.315×10^{-4}	2.34×10^{-9}
F5	8.675×10^{4}	6.345×10^{-4}	7.31×10^{-9}

Supplementary Figure 2. Kinetic characterization of 24 additional high-affinity nanobody variants.

(A) BLI sensorgrams. Bio-Layer Interferometry (BLI) measurements for 24 unique nanobody clones (D9 to F5) identified from the third biopanning round. (B) Summary of kinetic parameters.

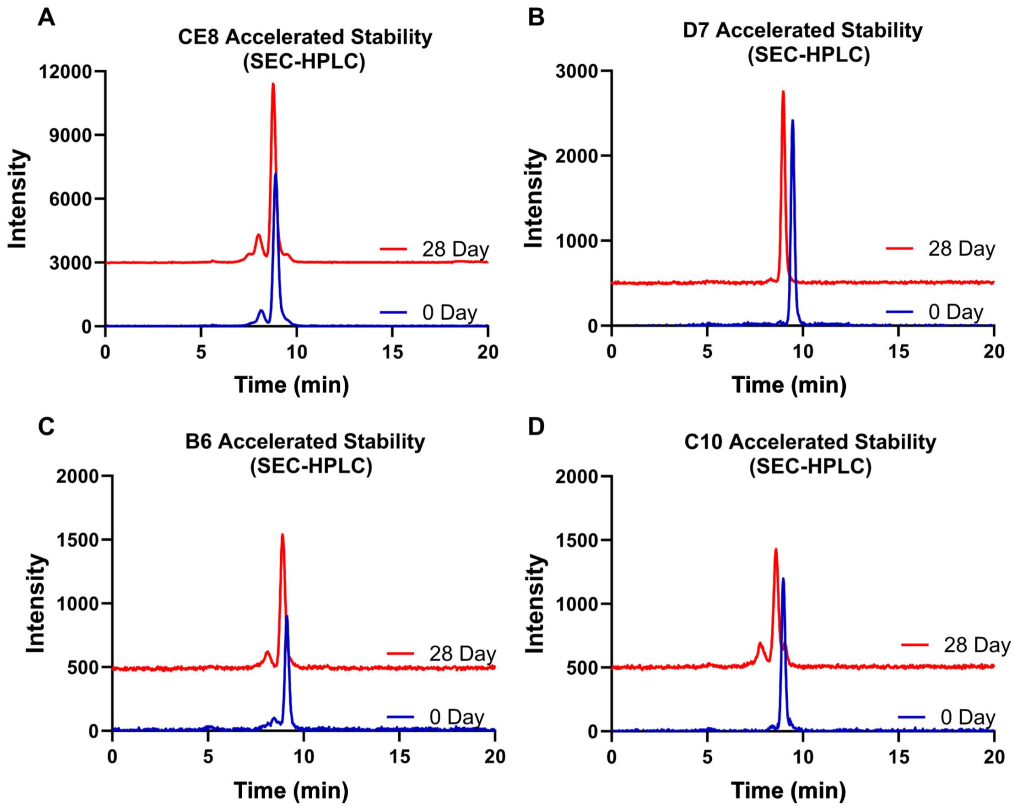

Supplementary Figure 3. Longitudinal SEC-HPLC analysis of nanobody variants under accelerated thermal stress.

(A – D) Individual SEC-HPLC chromatogram overlays for (A) CE8, (B) D7, (C) B6, and (D) C10 before (Day 0, blue lines) and after (Day 28, red lines) incubation at 37 ℃.

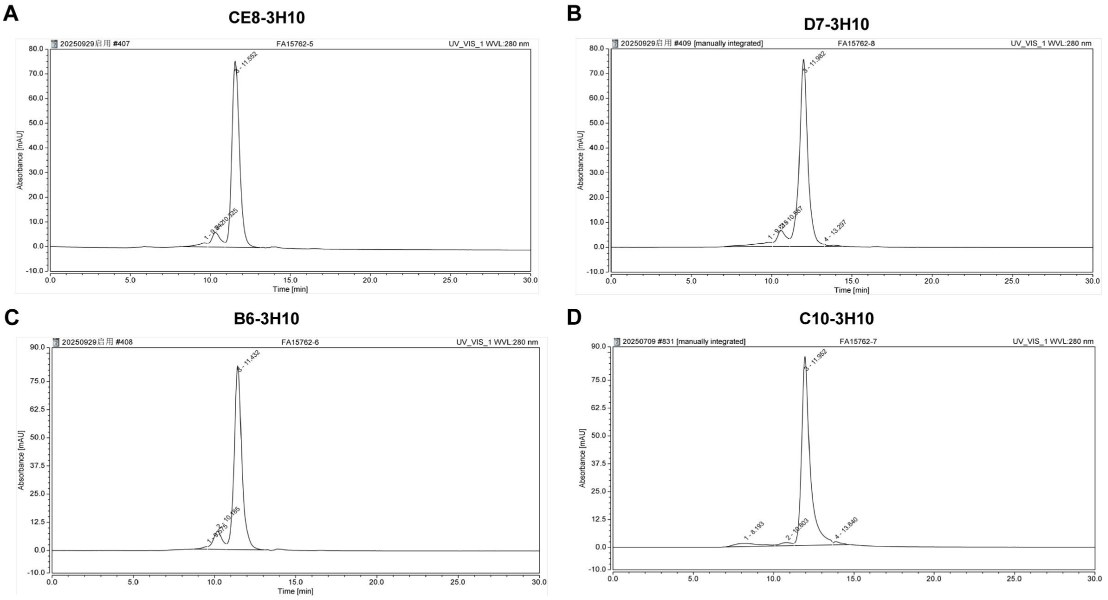

E

Bispecific antibodies binding CEA and HSA antigens sequentially

Antibody	Conc. of HSA (nM)	Binding signal 1 (nm)	Binding signal 2 (nm)	Signal 1 /Signal 2
CE8-3H10	100	0.188	0.200	0.940
	20	0.087	0.078	1.115
D7-3H10	100	0.172	0.183	0.940
	20	0.070	0.070	1.000
B6-3H10	100	0.153	0.167	0.916
	20	0.071	0.075	0.947
C10-3H10	100	0.174	0.191	0.911
	20	0.075	0.076	0.987

F

Bispecific antibodies binding CEA and MSA antigens sequentially

Antibody	Conc. of MSA (nM)	Binding signal 1 (nm)	Binding signal 2 (nm)	Signal 1 /Signal 2
CE8-3H10	100	0.157	0.173	0.908
	20	0.061	0.056	1.089
D7-3H10	100	0.188	0.196	0.959
	20	0.060	0.061	0.984
B6-3H10	100	0.149	0.154	0.968
	20	0.069	0.067	1.030
C10-3H10	100	0.188	0.189	0.995
	20	0.082	0.067	1.224

Supplementary Figure 4. Assessment of antibody homogeneity and simultaneous binding capability.
(A–D) Size-exclusion chromatography (SEC-HPLC) profiles of the purified bispecific nanobodies: CE8-3H10 (A), D7-3H10 (B), B6-3H10 (C), and C10-3H10 (D). The single dominant peak in each chromatogram indicates high monomeric purity (> 90%) and the absence of significant high-molecular-weight aggregates. (E–F) Tables summarizing the BLI binding signals for sequential antigen engagement. Bispecific antibodies at the indicated concentrations were exposed to CEA followed by HSA (E) or MSA (F). The "Signal 1/Signal 2" ratio represents the binding response of albumin in the presence of CEA relative to albumin alone. Ratios near 1.0 confirm that the two binding events are spatially independent and non-competitive.

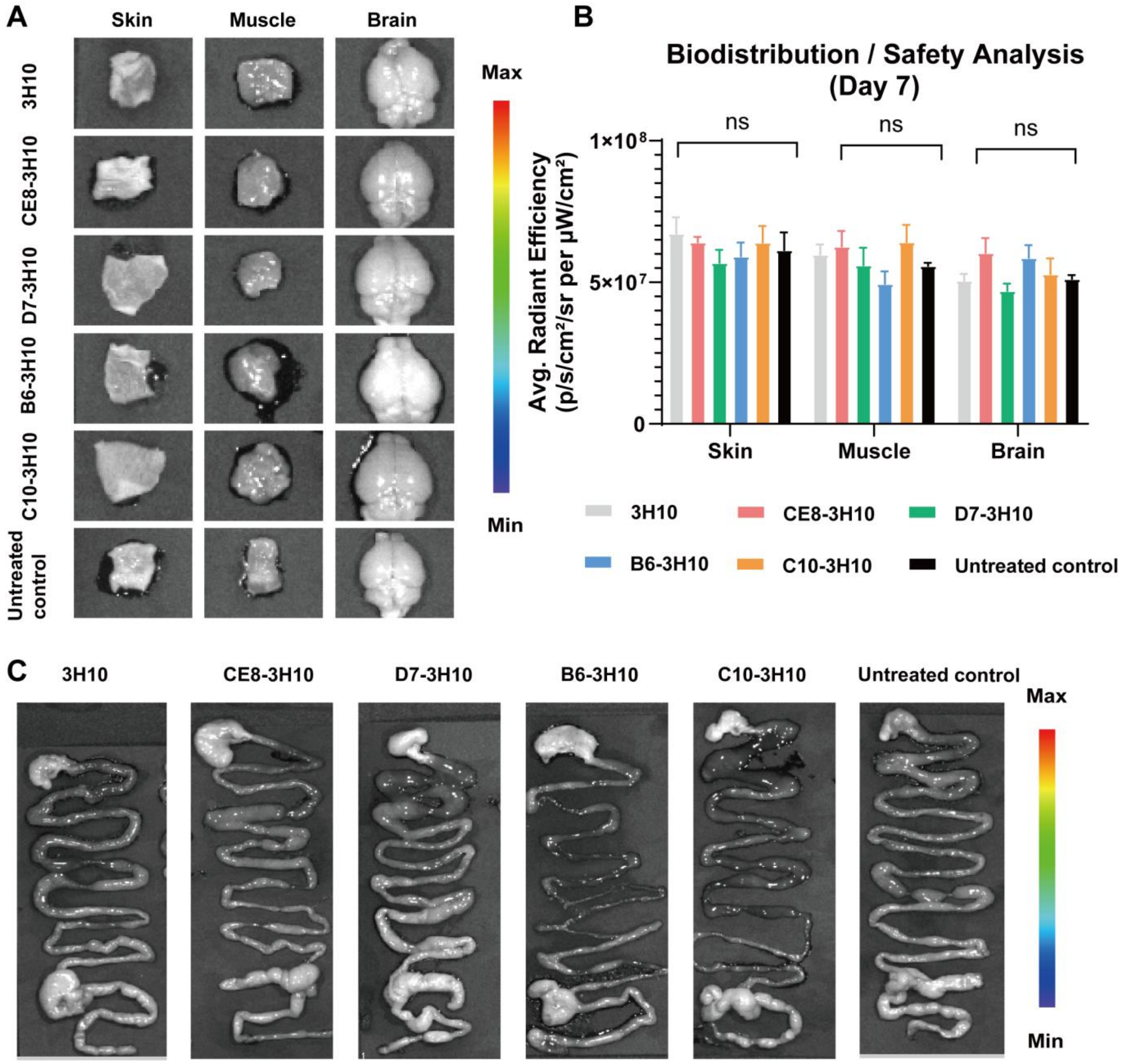

Supplementary Figure 5. Expanded systemic safety and biodistribution profiling.

(A) Representative *ex vivo* NIR fluorescence images of the skin, muscle, and brain harvested at the 168-hour endpoint (Day 7) following euthanasia. The panels compare the five experimental groups (3H10, CE8-3H10, D7-3H10, B6-3H10, and C10-3H10) against an untreated control to establish the baseline autofluorescence. (B) Quantitative analysis of the average radiant efficiency in the skin, muscle, and brain. No statistically significant accumulation was detected in any of the treatment groups compared to either the parental CE8-3H10 or the untreated control (*ns*, not significant). This confirms that ultra-high affinity engineering does not induce off-target retention in these non-target tissues. (C) *Ex vivo* NIR fluorescence imaging of the gastrointestinal tract (stomach and intestine) at Day 7. The consistent signal intensity across all groups, including the untreated control, indicates that the observed signals represent the endogenous background and that the engineered nanobodies do not exhibit gastrointestinal accumulation. Data information: In (B), data are presented as mean ± SD (n = 6 biologically independent animals per group). *ns*, not significant (P>0.05).

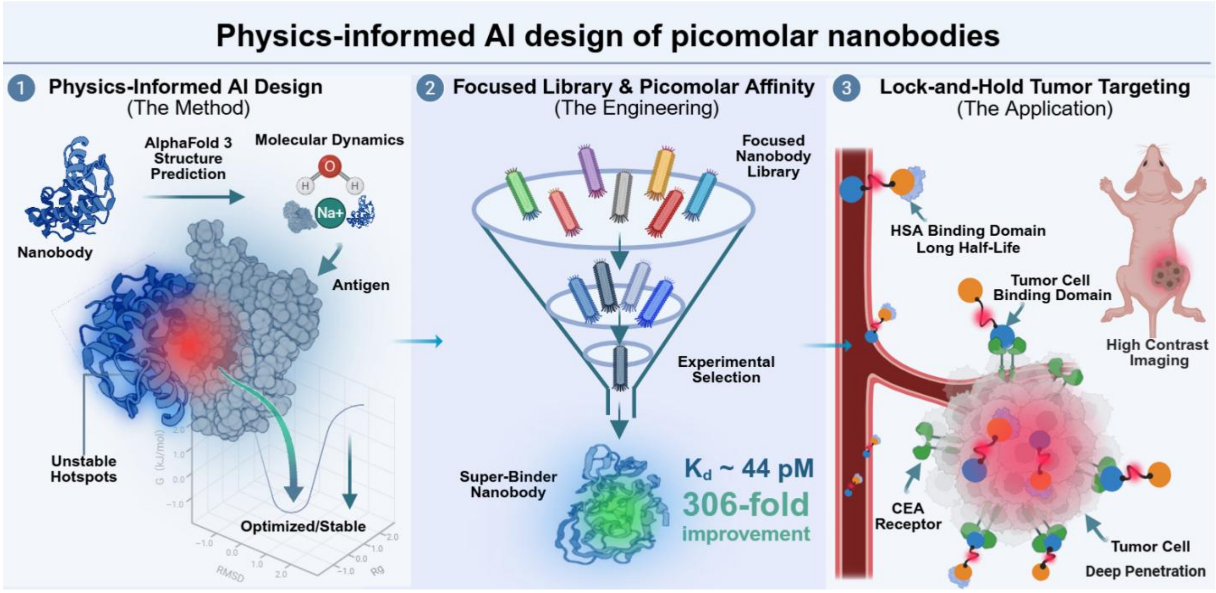

Supplementary Figure 6. Schematic illustration of the physics-informed AI framework for designing picomolar-affinity nanobodies.

The framework integrates thermodynamic optimization with evolutionary screening to generate super-binder nanobodies. The resulting bispecific constructs leverage albumin binding for extended circulation, achieving deep penetration and stable 'lock-and-hold' retention within dense solid tumors for precise targeting.

Table S1. Calculated binding free energy decomposition of CE8 variants via vd-MM/GBSA.

**Wild-Type vs Mutated Residues**	ΔG_{vdw}	ΔG_{elec}	ΔG_{GB}	ΔG_{SA}	ΔG_{total}
Wild-type (CE8)	-50.64	-46.26	72.76	-6.62	-30.76
S28N	-50.59	-45.86	72.25	-6.60	-30.80
S28T	-50.19	-48.39	74.06	-6.62	-31.14
Y29H	-47.76	-43.43	65.74	-6.05	-31.50
Y29F	-49.12	-43.63	67.89	-6.09	-30.95
F32Q	-50.21	-43.04	67.47	-6.81	-32.59
F32Y	-51.86	-51.95	77.04	-6.88	-33.65
N56S	-50.63	-43.73	70.12	-6.61	-30.85
N56T	-50.37	-41.56	66.89	-6.61	-31.65
N56Y	-50.65	-43.75	69.79	-6.62	-31.23
R102Q	-50.69	2.07	24.37	-6.70	-30.95
R102K	-50.59	-50.66	76.8	-6.71	-31.16
R102S	-49.39	0.43	24.06	-6.59	-31.49
S104R	-50.47	-88.13	113.54	-6.66	-31.72
S104H	-50.76	-46.41	72.58	-6.66	-31.25
S104Y	-50.63	-46.19	72.26	-6.65	-31.21
D108Q	-50.39	-86.11	111.41	-6.65	-31.74
D108S	-50.62	-85.09	110.67	-6.65	-31.69
D108Y	-50.90	-85.67	111.59	-6.65	-31.63
F110Q	-49.37	-43.11	68.21	-6.61	-30.88
F110S	-50.24	-45.54	70.84	-6.67	-31.61
F110Y	-50.19	-42.94	68.77	-6.63	-30.99

* Note: All energy values are reported in kcal/mol. ΔG_{vdw}, van der Waals; ΔG_{elec}, electrostatic; ΔG_{GB}, polar solvation; ΔG_{SA}, non-polar solvation energy

Table S2. Summary of the precision degenerate codon design for the focused nanobody library.

Hotspot	Target Amino Acids	Design Strategy (IUPAC/Mixed)	Encoded Codons (Amino Acids)	By-product Analysis
S28	S, T, N	AVC	AGC(S), ACC(T), AAC(N)	None
Y29	Y, F, H	YAT(TTT)	CAT(H), TAT(Y), TTT(F)	None
F32	F, Q, Y	TWT(CAG)	TTT(F), TAT(Y), CAG(Q)	None
N56	N, Y, S, T	TMT(AMC)	TAT(Y),TCT(S), AAC(N), ACC(T)	None
R102	R, K, S, Q	MGC(MAA)	CGC(R),AGC(S),AAA(K), CAA(Q)	None
S104	S, Y, H, R	YAC(AGK)	TAC(Y),CAC(H),AGG(R), AGT(S)	None
D108	D, S, Q, Y	TMT(GAC/CAG)	TAT(Y),TCT(S),GAC(D), CAG(Q)	None
F110	F, S, Y, Q	THT(CAG)	TTT(F),TCT(S), TAT(Y), CAG(Q)	None

* Note: Parentheses indicate a primer-mixing strategy (split-synthesis) at the specific site to ensure 100% fidelity while eliminating premature stop codons. IUPAC codes: R=A/G; Y=C/T; M=A/C; K=G/T; W=A/T; H=A/C/T; V=A/C/G.

Table S3. Computational resources and execution time for the Physics-Informed AI pipeline.

Optimization Step	Tools & Software	Estimated Time	Key Output / Deliverable
Structural Modeling	AlphaFold 3, HADDOCK2.4	24 Hours	Ensemble of 100 high-confidence models
Equilibration & MD Simulation	GROMACS 2024.2	24-72 Hours	100 ns trajectories (in triplicates)
CDR Region Definition	IMGT	1 Hour	Definition of CDR1, CDR2, and CDR3
Interface Residue Identification	PyMOL, PDBePISA	1-2 Hours	Identification of paratope residues within 6 Å
Decomposition & Hotspot Discovery	MM/GBSA	6-8 Hours	Identification of 14 energetic bottlenecks
In Silico Mutagenesis Scanning	MM/GBSA	12-24 Hours	Predicted ΔG_{bind} for all variants
Total		~68-131 Hours	Final optimized mutation combinations

Table S4. Predicted MHC-II-restricted T-cell epitopes across HLA-DRB1 alleles.

Antibody	DRB1-0101SB/WB	DRB1-0401SB/WB	DRB1-0405SB/WB	DRB1-0701SB/WB	DRB1-0901SB/WB	Total SB
CE8	4 / 0	7 / 4	3 / 0	3 / 0	5 / 0	22
C10	4 / 0	7 / 4	3 / 0	3 / 0	5 / 0	22
B6	0 / 0	3 / 1	0 / 0	0 / 0	0 / 0	3
D7	0 / 0	0 / 1	0 / 0	0 / 0	0 / 0	0

*Note: SB (Strong Binder) defined as %Rank_EL ≤ 0.5%; WB (Weak Binder) defined as 0.5% < %Rank_EL ≤ 2.0%. A total of ten HLA-DRB1 alleles were tested; alleles with no SB/WB detected in any variant are omitted for brevity.
